# Impact of dexmedetomidine on the incidence of delirium in elderly patients after cardiac surgery: A randomized controlled trial

**DOI:** 10.1371/journal.pone.0170757

**Published:** 2017-02-09

**Authors:** Xue Li, Jing Yang, Xiao-Lu Nie, Yan Zhang, Xue-Ying Li, Li-Huan Li, Dong-Xin Wang, Daqing Ma

**Affiliations:** 1 Department of Anesthesiology and Critical Care Medicine, Peking University First Hospital, Beijing, China; 2 Department of Anesthesiology, Fuwai Hospital, National Center for Cardiovascular Diseases, Chinese Academy of Medical Sciences and Peking Union Medical College, Beijing, China; 3 Center for Clinical Epidemiology & Evidence-Based Medicine, Beijing Children’s Hospital, Capital Medical University, Beijing, China; 4 Department of Biostatistics, Peking University First Hospital, Beijing, China; 5 Section of Anaesthetics, Pain Management and Intensive Care, Department of Surgery and Cancer, Imperial College London, Chelsea and Westminster Hospital, London, United Kingdom; Universidade do Extremo Sul Catarinense, BRAZIL

## Abstract

**Background:**

Delirium is a frequent complication after cardiac surgery and its occurrence is associated with poor outcomes. The purpose of this study was to investigate the impact of perioperative dexmedetomidine administration on the incidence of delirium in elderly patients after cardiac surgery.

**Methods:**

This randomized, double-blinded, and placebo-controlled trial was conducted in two tertiary hospitals in Beijing between December 1, 2014 and July 19, 2015. Eligible patients were randomized into two groups. Dexmedetomidine (DEX) was administered during anesthesia and early postoperative period for patients in the DEX group, whereas normal saline was administered in the same rate for the same duration for patients in the control (CTRL) group. The primary endpoint was the incidence of delirium during the first five days after surgery. Secondary endpoints included the cognitive function assessed on postoperative days 6 and 30, the overall incidence of non-delirium complications within 30 days after surgery, and the all-cause 30-day mortality.

**Results:**

Two hundred eighty-five patients were enrolled and randomized. Dexmedetomidine did not decrease the incidence of delirium (4.9% [7/142] in the DEX group vs 7.7% [11/143] in the CTRL group; OR 0.62, 95% CI 0.23 to 1.65, p = 0.341). Secondary endpoints were similar between the two groups; however, the incidence of pulmonary complications was slightly decreased (OR 0.51, 95% CI 0.26 to 1.00, p = 0.050) and the percentage of early extubation was significantly increased (OR 3.32, 95% CI 1.36 to 8.08, p = 0.008) in the DEX group. Dexmedetomidine decreased the required treatment for intraoperative tachycardia (21.1% [30/142] in the DEX group vs 33.6% [48/143] in the CTRL group, p = 0.019), but increased the required treatment for postoperative hypotension (84.5% [120/142] in the DEX group vs 69.9% [100/143] in the CTRL group, p = 0.003).

**Conclusions:**

Dexmedetomidine administered during anesthesia and early postoperative period did not decrease the incidence of postoperative delirium in elderly patients undergoing elective cardiac surgery. However, considering the low delirium incidence, the trial might have been underpowered.

**Trial Registration:**

ClinicalTrials.gov NCT02267538

## Introduction

Delirium is a common complication after cardiac surgery, with reported incidences varied from 3% to 47% [1]. The occurrence of delirium after cardiac surgery is associated with worse outcomes; these include increased rate of complications, prolonged duration of mechanical ventilation, prolonged length of stay in ICU and hospital, and increased medical expenses during hospitalization, as well as increased readmission rate, compromised long-term cognitive function, decreased physical ability and life quality, and elevated long-term mortality after hospital discharge [2, 3]. The pathogenesis of postoperative delirium is not fully understood whilst previous studies demonstrated that deep anesthesia during surgery [4, 5] and use of high doses opiates and/or sedatives after surgery [6, 7] are important predisposing factors.

Dexmedetomidine is a highly selective alpha 2-adrenoceptor agonist with anxiolytic, sedative, and analgesic properties [8], and is widely used as an adjuvant during general anesthesia and for sedation during mechanical ventilation after surgery [9]. Studies showed that, for patients undergoing cardiac surgery, use of dexmedetomidine during general anesthesia decreased the consumption of opioid and other anesthetic drugs [10,11], and use of dexmedetomidine for sedation after surgery shortened the duration of mechanical ventilation and decreased the incidence of delirium [12,13]. We hypothesized that use of dexmedetomidine as an anesthetic adjuvant during cardiac surgery decreased the incidence of delirium, possibly by sparing the consumption of general anesthetics. Evidence in this aspect was still lacking. The purpose of this study was to investigate the effect of dexmedetomidine administered during and shortly after cardiac surgery on the incidence of delirium in elderly patients (age of 60 years or over).

## Methods

### Study design

This was a randomized, double-blind, and placebo-controlled two-center trial. The study protocol (S1 Protocol) was approved by the Ethics Committees of Peking University First Hospital (2014–805) and Beijing Fuwai Hospital (2015–646), and was registered with ClinicalTrials.gov number, NCT02267538. Written informed consent was obtained from each patient or, if the patient could not provide informed consent, from the surrogate of the patient.

### Participants

Potential participants were screened the day before surgery. The inclusion criteria were elderly patients (age ≥ 60 years) who were scheduled to undergo elective coronary artery bypass graft and/or valve replacement surgery. Patients who met any of the following criteria were excluded: (1) previous history of schizophrenia, epilepsy, Parkinson disease, or severe dementia; (2) inability to communicate because of severe visual/auditory dysfunction or language barrier; (3) previous history of functional neurosurgery or brain injury; (4) preoperative sick sinus syndrome, severe bradycardia (heart rate < 50 beat per minute), second-degree or above atrioventricular block without pacemaker; (5) severe hepatic insufficiency (Child-Pugh grades C); or (6) severe renal insufficiency (requirement of renal replacement therapy); (7) patient refused to participate in the study.

### Randomization, intervention and anesthesia management

Center-stratified randomization with a block size of 4 was done using the SAS statistical package version 9.3 (SAS Institute, Cary, NC, USA) by an independent biostatistician. Study drugs, either dexmedetomidine hydrochloride 200 μg /2 ml or 0.9% sodium chloride 2 ml, were provided as clear aqueous solutions in the same 3 ml bottles (manufactured by Jiangsu Hengrui Pharmaceutical Co., Ltd., China) and encoded according to the randomization order by an independent pharmacist who did not participated in the rest of the study. The randomization results were then sealed in sequentially numbered letters and stored at the site of investigation until the end of the study.

During the study period, a study coordinator distributed the study drugs according to the recruitment ranking number and recorded the study drug codes in the Case Report Form. Study drugs were diluted with normal saline to 50 ml before administration, for dexmedetomidine the final concentration was 4 μg/ml. In the operating room, study drug infusion was started once the intravenous access was established, firstly at a rate of [0.9 × kg] ml/h (i.e., 0.6 μg/kg for dexmedetomidine) for 10 minutes, then at a rate of [0.1 × kg] ml/h (i.e., 0.4 μg·kg^-1^·h^-1^ for dexmedetomidine) until the end of surgery. After surgery, study drug infusion was continued at a rate of [0.025 × kg] ml/h (i.e., 0.1 μg·kg^-1^·h^-1^ for dexmedetomidine) until the end of mechanical ventilation. All investigators, healthcare team members and patients were blinded to the treatment group assignment throughout the study period.

Premedication could be administered before anesthesia according to patients’ status. Anesthesia was induced with midazolam, etomidate, sufentanil, and propofol, and was maintained with sufentanil and propofol infusion and/or sevoflurane inhalation, with a target bispectral index (BIS) between 40 and 60. During surgery, hemodynamics was carefully maintained by adjusting fluid infusion and using inotropic and/or vasoactive drugs. All patients were transferred to the intensive care unit (ICU) after surgery. Patient-controlled analgesia pump was applied whenever possible. For patients who required additional analgesia, opiates were administered either intravenously or orally. For patients who required sedation (i.e., patients undergoing mechanical ventilation), propofol intravenous infusion was the first choice, with a target Richmond Agitation Sedation Scale (RASS) [14] maintained between -2 and +1. Midazolam was administered when necessary. For all enrolled patients, penehyclidine hydrochloride and scopolamine were prohibited, and open-labled dexmedetomidine was not allowed during the whole study period.

### Outcome assessment and follow-up schedule

Prior to the study, two investigators (XL and YZ) who performed interview and delirium assessments were trained to follow standard procedures and to use the Confusion Assessment Method (CAM) and the CAM for the Intensive Care Unit (CAM-ICU) by a psychiatrist. Both CAM and CAM-ICU make the diagnosis according to four features of delirium: (1) acute onset of mental status changes or a fluctuating course; (2) inattention; (3) disorganized thinking; and (4) altered level of consciousness. To have delirium diagnosed, a patient must display features 1 and 2, with either 3 or 4 [15,16].

Before surgery, detailed baseline data including demographics, comorbidities, medication, laboratory test results, diagnosis, and ASA classification were collected. Preoperative evaluations were performed with Mini-Mental State Examination (MMSE, scores range from 0 to 30, with higher scores indicating better cognitive function), Hospital Anxiety and Depression Scale (HADS, scores range from 0 to 42, with higher scores indicating more severe anxiety and depression), Barthel Index (BI, scores range from 0 to 100, with higher scores indicating better daily life activity), and CAM.

Intraoperative data including type of surgery, duration of anesthesia and surgery, names and doses of anesthetic drugs, use and duration of cardiopulmonary bypass and aortic cross-clamping, transfusion of blood products, and fluid balance were recorded. The average BIS values from 5 minutes after anesthesia induction to the end of surgery were acquired. After ICU admission, heart rate, blood pressure, respiratory rate, pulse oxygen saturation, urine output and fluid infusion were recorded hourly. Use of analgesics, sedatives, vasoactive drugs and occurrence of adverse events were documented. Acute Physiology and Chronic Health Evaluation II (APACHE II, scores range from 0 to 71, with higher scores indicating more severe illness) was scored according to the worst condition during the first 24 hours in the ICU.

Delirium was assessed daily (from 8 to 10 am) during postoperative days 1 to 5. Non-intubated patients were assessed with the CAM; intubated patients were assessed with the CAM-ICU. Before delirium assessment, the level of sedation/agitation was evaluated with the Richmond Agitation Sedation Scale (RASS) [14]. If the patient was deeply sedated or was unarousable (-4 or -5 on the RASS), delirium assessment was stopped and the patient was noted as comatose. If the RASS was above -4 (-3 through +5), delirium assessment was performed. For patients who were discharged or died before postoperative day 5, the results of last delirium assessment were used as the missing data. After the completion of daily delirium assessment, the intensity of pain both at rest and with coughing was evaluated using the numeric rating scale (NRS, an 11-point scale where 0 indicated no pain and 10 indicated the worst pain) and the subjective sleep quality was also evaluated using the NRS (an 11-point scale where 0 indicated the best sleep quality and 10 indicated the worst sleep experience).

On postoperative day 6, cognitive function was reevaluated using the MMSE; the duration of mechanical ventilation, the length of stay in the ICU and hospital after surgery, and the occurrence of non-delirium complications were recorded. Non-delirium complications were defined to be any medical conditions other than delirium that required therapeutic intervention and occurred within 30 days after surgery. On postoperative day 30, patients were followed-up by telephone interview; any conditions that led to re-hospitalization were documented; cognitive function was evaluated using Chinese version of Telephone Interview for Cognitive Status-modified scale (m-TICS, scores range from 0 to 48, with higher scores indicating better cognitive function).

The primary endpoint was the incidence of delirium within the first five days after surgery. Secondary endpoints included the cognitive function assessed on postoperative days 6 and 30, the overall incidence of non-delirium complications within 30 days after surgery, and the all-cause 30-day mortality. Other predefined endpoints included the severity of pain and subjective sleep quality during the first 5 days after surgery, the duration of mechanical ventilation, the length of stay in ICU and hospital after surgery, and the re-hospitalization rate within 30 days after surgery.

### Statistical analysis

#### Sample size estimation

According to published data and our own results, we assumed that the incidence of delirium after cardiac surgery was 30% in the CTRL group patients [1,17]. A meta-analysis showed that the incidence of delirium was reduced by 63% (RR 0.36, 95% CI 0.21 to 0.64) when dexmedetomidine was used for sedation in patients after cardiac surgery [12]. We conservatively assumed that the incidence of delirium would be reduced by 50% in the dexmedetomidine group. With significance and power set at 0.05 (two-sided) and 80% respectively, the sample size required to detect differences was 236 patients. Taking into account a drop-out rate of about 17%, we planned to enroll 284 patients. Sample size calculation was performed with the PASS 11.0 software (StataCorp. LP, College Station, TX).

#### Outcome analyses

Continuous variables with normal distribution were analyzed using the unpaired t-test. Continuous variables with abnormal distribution or ordinal data were analyzed with Mann-Whitney U test. Median differences were calculated with the Hodges-Lehmann estimator. Categorical variables were analyzed using the Chi-squared test, continuity correction Chi-squared test or Fisher exact test, with odds ratio (OR) calculated by logistic analysis. Time-to-event results were analyzed using the Kaplan-Meier survival analysis, with the difference between groups tested by the log-rank test and hazard ratio (HR) calculated by Cox regression analysis.

Outcome and safety data were analyzed in the intent-to-treat population. We also did per-protocol analysis for the primary endpoint; patients who were randomized but did not complete study drug administration were excluded from per protocol analysis. Statistical analysis was performed with SAS statistical package (version 9.3, SAS Institute, Cary, NC, USA) by statisticians in Peking University First Hospital and Beijing Children’s Hospital, with two-sided P values of less than 0.05 as statistical significance. This manuscript adhered to the applicable CONSORT guideline.

## Results

### Patient population

**Fig 1:**
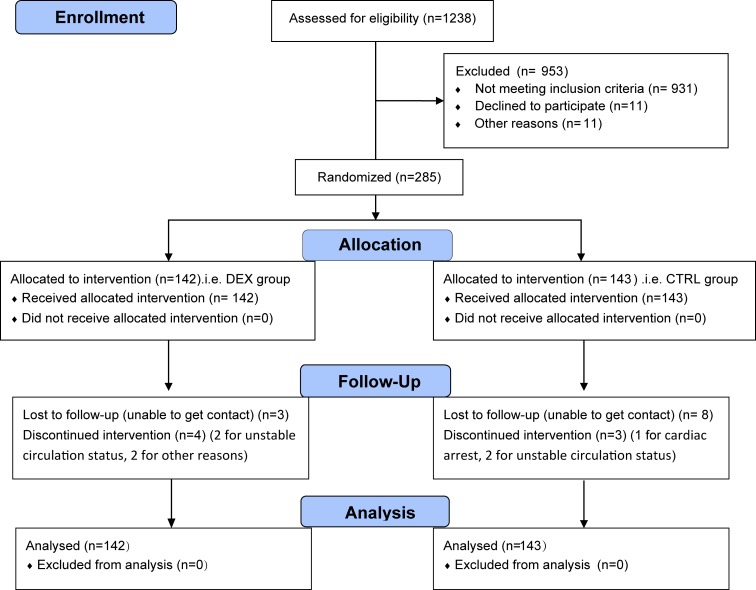
Flow Diagram of the study. DEX, dexmedetomidine; CTRL, control; ITT, Intention-to-Treat.

From December 1, 2014 to July 19, 2015, 1238 patients were screened for eligibility; 307 patients met the inclusion/exclusion criteria; 285 patients gave consents and were randomized into the study, among them 142 in the DEX group and 143 in the CTRL group. During the study period, postoperative study drug infusion was not completed in 7 patients (4 cases in the DEX group and 3 cases in the CTRL group) because of cardiac arrest, unstable circulation status or other reasons in the ICU; 6 patients died within 30 days after surgery (2 cases in the DEX group and 4 cases in the CTRL group). At postoperative day 30, 11 patients (3 cases in the DEX group and 8 cases in the CTRL group) were lost to follow-up. Each of these patients was included in the final Intention-to-Treat analysis (Fig 1).

The percentage of patients with baseline hyperlipidemia was lower in the DEX group than in the CTRL Group (p = 0.005). During intraoperative period, the median average BIS value was lower in the DEX group than in the CTRL group (median 42 [interquartile range 38 to 46] with DEX vs 44 [41 to 49] with CTRL, p < 0.001). There were no significant differences between the two groups regarding other baseline parameters and perioperative variables (Tables 1 and 2).

**Table 1:** Baseline data.

	CTRL group (n = 143)	DEX group (n = 142)
Age, year	67.5 ± 5.3	66.4 ± 5.4
Male gender	102 (71.3%)	95 (66.9%)
BMI, kg/m^2^	24.9 ± 2.8	25.3 ± 3.4
Education, year	9 (5 to 10)	9 (5 to 12)
Comorbidities		
Hypertension	91 (63.6%)	89 (62.7%)
Arrhythmia	29 (20.3%)	32 (22.5%)
Stroke	33 (23.1%)	26 (18.3%)
COPD	4 (2.8%)	7 (4.9%)
Diabetes Mellitus	49 (34.3%)	43 (30.3%)
Hyperlipidemia^*a*^	61 (42.7%)	38 (26.8%)
Chronic kidney disease^*b*^	7 (4.9%)	1 (0.7%)
Tumor^*c*^	3 (2.1%)	7 (4.9%)
Thyroid disease^*d*^	3 (2.1%)	4 (2.8%)
Smoking^*e*^	66 (46.2%)	51 (35.9%)
Acute myocardial infarction	11 (7.7%)	17 (12.0%)
Preoperative medications		
CCB	45 (31.5%)	33 (23.2%)
ACEI/ARB	46 (32.2%)	50 (35.2%)
β-blocker	66 (46.2%)	63 (44.4%)
Statins	75 (52.4%)	63 (44.4%)
Antiplatelet drugs^*f*^	82 (57.3%)	85 (59.9%)
EuroSCORE	3 (2 to 5)	3 (2 to 5)
History of surgery	52 (36.4%)	55 (38.7%)
History of general anesthesia	24 (16.8%)	24 (16.9%)
NYHA function class^*g*^	(n = 132)	(n = 125)
I	2 (1.5%)	2 (1.6%)
II	92 (69.7%)	87 (69.6%)
III	36 (27.3%)	33 (26.4%)
IV	2 (1.5%)	3 (2.4%)
LVEF%	64.8 (58.2 to 70.6)	64.4 (56.2 to 70.4)
ASA Classification		
II	2 (1.4%)	1 (0.7%)
III	129 (90.2%)	128 (90.1%)
IV	12 (8.4%)	13 (9.2%)
MMSE score	29 (28 to 30)	29 (28 to 30)
HAD score	8 (4 to 12)	8 (4 to 12)
BI score	100 (95 to 100)	100 (95 to 100)
Delirium	0 (0.0%)	0 (0.0%)

Results are presented as mean ± standard deviation, number (%) or median (interquartile range).

BMI, body mass index; COPD, chronic obstructive pulmonary disease; CCB, calcium channel blocker; ACEI/ARB, angiotensin converting enzyme inhibitor/angiotensin receptor blocker; NYHA, New York Heart Association; EuroSCORE, European System for Cardiac Operative Risk Evaluation; MMSE, Mini-Mental State Examination; HAD, Hospital Anxiety and Depression Scale; BI, Barthel Index.

^*a*^ Serum total cholesterol > 5.18 mmol/L, triglyceride > 1.7mmol/L, or low density lipoprotein > 3.37 mmol/L

^*b*^ Diagnosed according to KDIGO criteria

^*c*^ Including breast cancer, lung cancer, gastric cancer, rectal cancer, and atrial myxoma

^*d*^ Including thyroid nodule/adenoma/cancer, hyperthyroidism/hypothyroidism, and history of thyroid surgery

^*e*^Smoking regularly for more than 1 year

^*f*^ Including aspirin, clopidogrel, and ticagrelor

^*g*^ exclude patients with acute myocardial infarction.

**Table 2:** Intra- and postoperative data.

	CTRL group (n = 143)	DEX group (n = 142)	P value
Premedication			
Morphine^*a*^, mg	10 (5 to 10)	10 (5 to 10)	0.500
Estazolam^*b*^, mg	2 (1 to 2)	2 (1 to 2)	0.569
Duration of anesthesia, min	256 (217 to 300)	250 (220 to 294)	0.889
Dose of anesthetics			
Midazolam^*c*^, mg	3 (2 to 5)	3 (2 to 5)	0.171
Propofol, mg	600 (400 to 920)	520 (300 to 825)	0.098
Etomidate^*d*^, mg	20 (20 to 20)	20 (20 to 20)	0.702
Sufentanil^*e*^, μg	200 (150 to 300)	225 (140 to 300)	0.967
Fentanyl^*e*^, mg	1.88 (1.70 to 2.03)	1.55 (1.50 to 1.60)	0.133
Average BIS value^*f*^	44 (41 to 49) (n = 128)	42 (38 to 46) (n = 130)	< 0.001
Duration of surgery, min	185 (157 to 235)	180 (159 to 225)	0.651
Type of surgery			0.731
CABG	103 (72.0%)	101 (71.1%)	
Valve replacement	19 (13.3%)	23 (16.2%)	
CABG and valve replacement	21 (14.7%)	18 (12.7%)	
Use of CPB	82 (57.3%)	82 (57.7%)	0.945
Duration of CPB^*g*^, min	101 (81 to 130)	105 (84 to 129)	0.979
Duration of AOC^*g*^, min	72 (49 to 92)	71 (59 to 91)	0.477
Duration of hypothermia^*g*^, min	60 (40 to 83)	59 (48 to 77)	0.395
APACHE II score	8 (7 to 11)	8 (6 to 10)	0.099
Use of PCA	15 (10.5%)	20 (14.1%)	0.355
Additional sedatives/analgesics			
Propofol^*h*^, mg	700 (400 to 1200)	600 (400 to 1000)	0.299
Benzodiazepines^*i*^	61 (42.7%)	76 (53.5%)	0.067
Analgesics intravenously^*j*^	10 (7.0%)	9 (6.3%)	0.825
Analgesics orally^*k*^	74 (51.7%)	87 (61.3%)	0.105
Total duration of study drug Infusion, h	18.7 (13.3 to 23.5)	18.7 (13.1 to 21.6)	0.279

Results are presented as mean ± standard deviation, number (%) or median (interquartile range).

BIS, bispectral index; CABG, coronary artery bypass graft; CPB, cardiopulmonary bypass; AOC, aortic cross clamping; APACHE, acute physiology and chronic health evaluation; PCA, patient-controlled analgesia.

^*a*^ Data of 130 patients (63 in CTRL group and 67 in DEX group) who received morphine as premedication

^*b*^ data of 261 patients (128 in CTRL group and 133 in DEX group) who received estazolam as premedication

^*c*^ data of 280 patients (141 in CTRL group and 139 in DEX group) who received midazolam during anesthesia

^*d*^ data of 125 patients (61 in CTRL group and 64 in DEX group) who received etomidate during anesthesia

^*e*^ data of 279 patients (139 in CTRL group and 140 in DEX group) who received sufentanil and 6 patients (4 in CTRL group and 2 in DEX group) who received fentanyl during anesthesia

^*f*^ average BIS value from 5 minutes after anesthesia induction to the end of the surgery. Data were missed in 27 patients (15 in CTRL group and 12 in DEX group)

^*g*^ data of patients who underwent surgery with CPB

^*h*^ data of 173 patients (88 in CTRL group and 85 in DEX group) who received propofol during the postoperative period

^*I*^ Including diazepam, lorazepam, midazolam, and estazolam

^*j*^ Including morphine, fentanyl, and pethidine

^*k*^ Including oxycodone-acetaminophen tablet and morphine sulfate sustained-release tablet.

### Effectiveness analysis

There was no significant difference between the two groups regarding the incidence of delirium during the first 5 days after surgery (4.9% [7/142] in the DEX group vs 7.7% [11/143] in the CTRL group; odds ratio [OR] 0.62, 95% CI 0.23 to 1.65; p = 0.341) (Table 3). Per-protocol analysis also showed no significant difference in the incidence of delirium between groups (2.9% [4/138] in the DEX group vs 5.7% [8/140] in the CTRL group; OR 0.49, 95% CI 0.15 to 1.68; p = 0.257).

**Table 3:** Effectiveness outcomes.

	CTRL group (n = 143)	DEX group (n = 142)	Estimated effects	P value
**Primary outcome**				
Incidence of delirium within the first 5 days after surgery	11 (7.7%)	7 (4.9%)	0.62 (0.23 to 1.65)	0.341
**Secondary outcomes**				
MMSE score on postoperative day 6^*a*^	29 (27 to 30)	29 (27 to 30)	0 (0 to 0)	0.830
m-TICS score on postoperative day 30^*b*^	34 (32 to 36)	34 (32 to 37)	0 (-1 to 0)	0.405
Incidence of non-delirium complications within 30 days after surgery	84 (58.7%)	73 (51.4%)	0.74 (0.47 to 1.19)	0.214
Stroke	3 (2.1%)	3 (2.1%)	1.01 (0.20 to 5.08)	0.993
New onset arrythmia^*c*^	51 (35.7%)	42 (29.6%)	0.76 (0.46to 1.25)	0.274
Pulmonary complications^*d*^	27 (18.9%)	15 (10.6%)	0.51 (0.26 to 1.00)	0.050
Upper gastrointestinal bleeding^*e*^	4 (2.8%)	2 (1.4%)	0.50 (0.09 to 2.75)	0.423
Surgical bleeding^*f*^	3 (2.1%)	3 (2.1%)	1.01 (0.20 to 5.08)	0.993
Wound dehiscence or infection^*g*^	7 (4.9%)	11 (7.7%)	1.63 (0.61 to 4.34)	0.326
Acute kidney injury^*h*^	44 (30.8%)	37 (26.1%)	0.79 (0.47 to 1.33)	0.378
IABP assistance	12 (8.4%)	6 (4.2%)	0.48 (0.18 to 1.32)	0.156
All cause 30-day mortality^*i*^	4 (3.0%)	2 (1.4%)	0.48 (0.09 to 2.66)	0.399
**Other outcomes**				
Pain score after surgery, at rest^*j*^				
Day 1	3 (2 to 5)	3 (1 to 5)	0 (-1 to 0)	0.596
Day 2	3 (2 to 5)	4 (2 to 5)	0 (-1 to 0)	0.743
Day 3	2 (2 to 4)	3 (2 to 4)	0 (-1 to 0)	0.282
Day 4	2 (1 to 3)	2 (1 to 3)	0 (0 to 0)	0.368
Day 5	1(1 to 2)	2 (1 to 2)	0 (0 to 0)	0.397
Pain score after surgery, with coughing^*j*^				
Day 1	4 (3 to 6)	4 (2 to 6)	0 (-1 to 0)	0.486
Day 2	4 (3 to 6)	5 (3 to 6)	0 (-1 to 0)	0.414
Day 3	4 (2 to 5)	4 (3 to 5)	0 (-1 to 0)	0.187
Day 4	3 (2 to 4)	3 (2 to 4)	0 (-1 to 0)	0.127
Day 5	2 (2 to 3)	2 (2 to 3)	0 (0 to 0)	0.378
Subjective sleep quality after surgery, score^*k*^				
Day 1	2 (0 to 6)	2 (0 to 5)	0 (0 to 0)	0.777
Day 2	3 (2 to 6)	3 (2 to 6)	0 (-1 to 1)	0.919
Day 3	2 (2 to 4)	2 (2 to 5)	0 (0 to 0)	0.835
Day 4	2 (0 to 4)	2 (1 to 4)	0 (0 to 0)	0.321
Day 5	2 (0 to 2)	2 (0 to 3)	0 (0 to 0)	0.174
Incidence of coma within the first 5 days after surgery	2 (1.4%)	2 (1.4%)	1.00 (0.14 to 7.25)	0.994
Duration of delirium among patients who developed delirium, d	2 (1 to 4)	2 (1 to 3)	1 (-1 to 2)	0.328
Duration of mechanical ventilation, h (median [95% CI])	15.0 (13.9 to 16.1)	15.0 (13.7 to 16.3)	1.28 (1.01 to 1.63)	0.044
Extubation within 24 hours after surgery	122 (85.3%)	135 (95.1%)	3.32 (1.36 to 8.08)	0.008
Length of stay in ICU after surgery, h (median [95% CI])	46.0 (44.8 to 47.2)	45.0 (43.5 to 46.5)	1.03 (0.82 to 1.31)	0.788
Length of stay in hospital after surgery, d (median [95% CI])	9 (8 to 10)	9 (8 to 10)	0.97 (0.77 to 1.23)	0.826
Re-hospitalization within 30 days after surgery^*i*^	15 (11.1%)	16 (11.5%)	1.04 (0.49 to 2.20)	0.917

Results are presented as number (%) or median (interquartile range), unless otherwise indicated. Estimated effects were calculated in the direction of DEX vs. or minus CTRL; results are presented as odds ratio (95% CI), median difference (95% CI) or hazard ratio (95% CI).

MMSE, Mini-Mental State Examination; m-TICS, modified Telephone Interview for Cognitive Status; IABP, intra-aortic balloon pump; ICU, intensive care unit; CI, confidence interval.

^*a*^ 5 patients (3 in CTRL group and 2 in DEX group) did not complete the MMSE test on postoperative day 6

^*b*^ 17 patients (12 in CTRL group and 5 in DEX group) did not complete the m-TICS test on postoperative day 30

^*c*^ including new onset atrial fibrillation, II/III degree atrioventricular block, frequently premature ventricular contractions, premature ventricular contractions (bigeminy/trigeminy), ventricular tachycardia, and ventricular fibrillation

^*d*^ including pulmonary infection, pneumothorax and pleural effusion needed intervention (closed drainage of pleural cavity, needle/catheter insertion for drainage, lung recruitment maneuvers)

^*e*^ positive occult blood test results in gastric contents or stool with decreased hemoglobin, and requirement of blood transfusion

^*f*^ bleeding after surgery that required secondary surgical hemostasis

^*g*^ need further debridement and /or suturing

^*h*^ diagnosed according to KDIGO criteria

^*I*^ 11 patients (8 in CTRL group and 3 in DEX group) were lost to follow up

^*j*^ assessed with numeric rating scale where 0 = no pain and 10 = the most severe pain

^*k*^ assessed with numeric rating scale where 0 = the best possible sleep and 10 = the worst possible sleep.

The MMSE Score assessed on postoperative day 6 (median difference 0, 95% CI 0 to 0; p = 0.830) and m-TICS score assessed on postoperative day 30 (median difference 0, 95% CI -1 to 0; p = 0.405) were similar between the two groups; there were no significant differences between the two groups regarding the overall incidence of non-delirium complications within 30 days after surgery (OR 0.74, 95% CI 0.47 to 1.19; p = 0.214) and all-cause 30-day mortality (OR 0.48, 95% CI 0.09 to 2.66, p = 0.399), despite of the fact that the incidence of pulmonary complications tended to be lower in the DEX group than in the CTRL group (OR 0.51, 95% CI 0.26 to 1.00; p = 0.050) (Table 3).

The intensity of pain both at rest and with coughing as well as the subjective sleep quality at postoperative days 1 to 5 were similar between the two groups. The duration of mechanical ventilation was shorter in the DEX group than in the CTRL group (hazard ratio [HR] 1.28, 95% CI 1.01 to 1.63, p = 0.044); the percentage of patients extubated within 24 hours after surgery was higher in the DEX group than in the CTRL group (OR 3.32, 95% CI 1.36 to 8.08; p = 0.008). There were no significant differences between the two groups regarding the incidence of coma within 5 days after surgery, the duration of delirium, the lengths of stay in ICU and hospital after surgery, and the rate of re-hospitalization within 30 days after surgery (Table 3, S1 Dataset).

### Safety analysis

The percentage of patients requiring intraoperative treatment for tachycardia was lower in the DEX group than in the CTRL group (21.1% [30/142] in the DEX Group vs 33.6% [48/143] in the CTRL Group; p = 0.019); on the other hand, the percentage of patients requiring postoperative treatment for hypotension was higher in DEX group than in the CTRL group (84.5% [120/142] in the DEX Group vs 69.9% [100/143] in the CTRL Group; p = 0.003) (Table 4).

**Table 4:** Adverse events.

	CTRL group (n = 143)	DEX group (n = 142)	P value
Intraoperative period			
Bradycardia^*a*^	14 (9.8%)	21 (14.8%)	0.199
Treatment for bradycardia^*b*^	11 (7.7%)	19 (13.4%)	0.118
Hypotension^*c*^	10 (7.0%)	10 (7.0%)	0.987
Treatment for hypotension^*d*^	101 (70.6%)	112 (78.9%)	0.109
Treatment for tachycardia^*e*^	48 (33.6%)	30 (21.1%)	0.019
Treatment for hypertension^*f*^	45 (31.5%)	35 (24.6%)	0.200
Postoperative period			
Bradycardia^*a*^	6 (4.2%)	2 (1.4%)	0.287
Treatment for bradycardia^*b*^	5 (3.5%)	3 (2.1%)	0.727
Hypotension^*c*^	10 (7.0%)	5 (3.5%)	0.189
Treatment for hypotension^*d*^	100 (69.9%)	120 (84.5%)	0.003
Treatment for tachycardia^*e*^	11 (7.7%)	14 (9.9%)	0.518
Treatment for hypertension^*f*^	63 (44.1%)	48 (33.8%)	0.076

Results are presented as number (%).

^*a*^ Heart rate < 45 beat per minute or a decrease of more than 30% from baseline (average value in the ward), and lasting for at least 5 minutes

^*b*^ intrasvenous injection of atropine, and/or intravenous infusion of isoprenaline

^*c*^ a decrease of systolic blood pressure of more than 30% from baseline (average value in the ward) and lasting for at least 15 minutes

^*d*^ intravenous administration of vasoactive and/or inotropic drugs, including ephedrine, phenylephrine, aramine, dopamine, norepinephrine, epinephrine, dobutamine, milrinone, and/or levosimendan

^*e*^ intravenous administration of esmolol

^*f*^ intravenous administration of sodium nitroprusside, urapidil, nicardipine, and/or ditiazem.

## Discussion

Our results showed that for elderly patients undergoing elective cardiac surgery, dexmedetomidine administered during anesthesia and early postoperative period did not decrease the incidence of delirium within the first 5 days after surgery. The results of cognitive function assessed on postoperative days 6 and 30, the overall incidence of non-delirium complications within 30 days after surgery as well as the all-cause 30-day mortality were similar between the two groups. Dexmedetomidine infusion decreased the required treatment for intraoperative tachycardia but increased the required treatment for postoperative hypotension.

In the present study, the incidence of delirium in the CTRL group was 7.7%, much lower than our and others’ previous studies [17–21]. The reasons that led to the low delirium incidence in the current patient population may include the followings. Firstly, anticholinergic drugs were less used in the current study. For example, in our previous studies, 39.9% to 46.7% of patients received anticholinergics (especially penehyclidine hydrochloride, an anticholinergic with high blood-brain barrier penetrating property) [17, 18, 21], which might have led to higher incidence of delirium [22, 23]. In the present study, only 10.2% of patients received anticholinergics (only atropine for treatment of bradycardia). Secondly, benzodiazepines were much less used than previously. Benzodiazepines were often used as the main sedatives for patients in the ICU [13, 17, 24], which may increase the incidence of delirium [25, 26]. In the present study, propofol was used as the first choice sedative, which may be responsible for decreasing the incidence of delirium *per se* [27]. Thirdly, delirium-preventing measures were used more commonly in daily nursing practice, including reorientation, cognitive stimulating, sleep promotion, and hearing/vision aids [28]. Interestingly, some previous studies also reported low incidence of postoperative delirium [29, 30].

Dexmedetomidine has been used for sedation in mechanically ventilated patients after cardiac surgery. In two randomized trials, postoperative sedation with dexmedetomidine reduced the incidence of delirium (1/30 [3%] with dexmedetomidine vs 15/30 [50%] with propofol vs 15/30 [50%] with midazolam; P < 0.001 [13] and 16/91 [17.5%] with dexmedetomidine vs 29/92 [31.5%] with propofol; P = 0.028 [31], respectively). In two other randomized trials, however, use of dexmedetomidine sedation did not reduce the incidences of postoperative delirium despite trends of decrease (13/152 [8.6%] with dexmedetomidine vs 22/147 [15.0%] with morphine; P = 0.088 [32] and 1/32 [3.1%] with dexmedetomidine vs 5/32 [15.6%] with normal saline; P = 0.086 [33], respectively). It should be mentioned that, in the two later studies, droperidol or gabapentin was administered as or as part of the premedication [32,33], which might partially explain the reasons of low delirium incidence [34,35] and non-significant results (i.e., underpowered studies). In the present study, we administered a sedative dose dexmedetomidine during anesthesia but a sub-sedative dose dexmedetomidine during mechanical ventilation after surgery. Our results found that dexmedetomidine did not decrease the incident of delirium. Our neutral results were attributable to several reasons. Firstly, the protocol requested that the BIS value be maintained between 40 and 60 during surgery but the median average BIS value was lower in the DEX group than in the CTRL group, indicating a deeper level of anesthesia in the DEX group. Indeed, deep anesthesia and low BIS was reported to increase the incidence of delirium [4, 5]. Secondly, the dose and timing of postoperative DEX administration was not enough. In previous studies, sedative dose dexmedetomidine decreased the incidence of delirium after cardiac surgery [13, 31]. In a recent study of our group, the effect of low-dose dexmedetomidine (0.1 μg·kg^-1^·h^-1^) in decreasing delirium in postoperative ICU patients was dose-dependent [36]. Thirdly, although not statistically significant, slightly more benzodiazepines were administered in patients of the DEX group (P = 0.067 compared with CTRL group). This might have increased the occurrence of delirium and counteracted the effect of dexmedetomidine. Fourthly, the incidence of delirium in the CTRL group of our study was far lower than we expected (7.7% instead of 30%). Therefore, our pre-calculated sample size was insufficient to detect the difference between the two groups. It is true that if the sample size calculation is based on the current incidences of delirium in our present study, 1181 cases in each group are needed to verify the effect of dexmedetomidine.

Dexmedetomidine produces a mild analgesic effect by activating the α2 adrenoceptors in the spine cord [37,38]. Our study did not find significant differences in the NRS pain scores and the use of analgesics after surgery. This was very likely due to a large dose of opiates usually used during cardiac anesthesia which might have masked the analgesic effect of dexmedetomidine. Dexmedetomidine produces a hypnotic effect by activating the endogenous sleep-promoting pathway [39, 40] and improves the sleep quality in mechanically ventilated patients [41]. Our study did not find significant difference in the subjective sleep quality after surgery. As discussed above, this was possibly due to the short duration of dexmedetomidine infusion.

In the present study, use of dexmedetomidine did not decrease the overall incidence of non-delirium complications, but it tended to decrease the incidence of pulmonary complications after surgery. In accordance with this, the duration of mechanical ventilation was shortened and the percentage of extubation within 24 hours after surgery was increased in the DEX group. Preclinical studies demonstrated that dexmedetomidine suppressed oxidative stress and inflammatory response [42], and attenuated the degree of acute lung injury produced by remote organ ischemia/reperfusion [43]. It warrants future study to verify its pulmonary protection. Regarding safety, the use of dexmedetomidine decreased the required treatment for intraoperative tachycardia; however, it increased the required treatment for postoperative hypotension and therefore, its safety, other benefits and long-term outcome are subjected to study further.

There were several limitations in our study. Firstly, our study excluded patients who were unable to communicate due to visual/auditory dysfunction, language barrier or psychiatric disease, and those with severe hepatic or kidney disease. These patients were at a great increased risk of delirium. Therefore, the extrapolation of our study was limited. Secondly, delirium was assessed once daily in the present study. Considering the transient and fluctuating characteristics of delirium, we might have missed some positive cases. Lastly, because of the low incidence of delirium in the CTRL group, this trial was underpowered to detect difference between the two groups. A large sample size randomized trial is needed to further clarify the effects of perioperative dexmedetomidine (S1 CONSORT checklist).

## Conclusions

Our study showed that dexmedetomidine administered during anesthesia and early postoperative period did not decrease the incidence of postoperative delirium in elderly patients undergoing elective cardiac surgery. However, considering the low delirium incidence, the trial might have been underpowered. Dexmedetomidine decreased the required treatment for intraoperative tachycardia, but increased the required treatment for postoperative hypotension.

## Supporting information

S1 CONSORT Checklist CONSORT Checklist. (DOC)

S1 Dataset Relevant data underlying the main results. (XLS)

S1 Protocol Study protocol. (DOC)
