# Supplementary material for: Impact of dexmedetomidine on the incidence of delirium in elderly patients after cardiac surgery: A randomized controlled trial
Source: PLoS One. 2017 Feb 9;12(2):e0170757. doi: 10.1371/journal.pone.0170757 (PMC5300174; doi:10.1371/journal.pone.0170757)
Supplement: S1 Protocol — (DOC) [file pone.0170757.s003.doc]

**右美托咪定对心脏外科病人术后谵妄发生率的影响：**

**多中心、前瞻性、随机、双盲、安慰剂对照研究**

**研究方案**

**研究药物** 盐酸右美托咪定注射液

**研究负责人** 王东信教授

**研究设计**  王东信教授、李雪医师、李立环教授、马大青教授

**研究机构** 北京大学第一医院、中国医学科学院阜外心血院、英国伦敦帝国理工大学切尔西与威斯敏斯特医院

**版本号 V2.0**

**撰写日期 2014-09-10**

1. **研究摘要：**

**研究背景：**回顾性研究指出，围术期使用右美托咪定伴随心脏术后谵妄发生率降低。然而，此领域仍缺乏来自前瞻性随机对照研究的证据。

**研究目的：**探讨右美托咪定用于老年心脏手术病人全身麻醉及术后镇静辅助用药能否降低术后谵妄的发生率。

**研究设计**：双中心、前瞻、随机、双盲、安慰剂对照的研究。共设两个组，右美托咪定干预组及生理盐水对照组。

**研究中心：**北京大学第一医院及中国医学科学院阜外心血管病医院。

**研究人群：**60岁及以上的、拟行择期冠脉旁路移植术或瓣膜手术的284例老年病人。

**干预措施**：右美托咪定组在麻醉诱导前10min内给予负荷剂量右美托咪定0.6 μg/kg，继以0.4μg/kg/h速度维持输注至术毕，术后以0.1μg/kg/h速度输注直至停止机械通气；对照组则以相同的容量、速度输注生理盐水。

**主要研究终点：**术后前5天内谵妄的发生率。

**研究预期时限**：2-3年。

**研究注册：**[ClinicalTrials.gov](http://www.clinicaltrials.gov/), number NCT02267538.

**2.背景**

谵妄是一种急性暂时性中枢神经系统功能异常，以认知功能障碍、意识水平下降、注意力不能集中、精神活动力下降和睡眠-觉醒周期紊乱为特征，常发生在术后几小时至几天之内，并在1天之内有明显波动[1]。谵妄是心脏外科手术后常见的并发症，报告的发生率为3-47%[2]。研究显示，谵妄发生伴随ICU停留时间和住院时间延长[3, 4]、住院期间并发症发生率增加[5]及医疗费用增多[6, 7]；此外，谵妄发生还伴随术后远期预后恶化，包括认知功能下降[8]、生存质量降低[9]，及死亡率增加[10, 11]。可见，围术期谵妄的发生与否将对病人的预后产生显著影响。

术后谵妄发生的机制仍未完全明确，但术后谵妄的出现通常是多种因素共同作用的结果。既往研究提示深麻醉[12, 13]、严重手术应激[14, 15]、剧烈疼痛[16, 17]、大剂量阿片类药物的使用[18, 19]、机械通气时间延长[20, 21]、睡眠剥夺[22, 23]等均与谵妄的发生有关。故理论上，在保障足够麻醉深度的前提下减少全麻药的使用、减轻炎症反应、降低交感应激、缓解疼痛以及提高睡眠质量的措施应能够降低谵妄的发生率。

右美托咪定是一种高选择性α2-肾上腺素能受体激动剂，具有中枢抗交感和抗焦虑作用，能产生近似自然睡眠的镇静作用，同时具有一定的镇痛、利尿作用，对呼吸无明显抑制，目前正广泛应用于全麻术中或术后镇静[24]。已有研究表明，右美托咪定用作全麻术中辅助用药可减少病人对阿片类药物及其他镇静药的需要量，并能减轻应激程度[25-27]。右美托咪定用于术后镇静，亦能减少其他镇静药及镇痛药的使用，提高镇痛效果、改善睡眠质量并降低谵妄的发生率[28-31]。既往回顾性研究表明，对于心脏手术病人，围术期使用右美托咪定能够降低谵妄发生率、非谵妄并发症发生率以及1年死亡率[31-33]。然而，目前在此方面，仍缺少来自前瞻性随机对照试验的证据。

我们推测右美托咪定用于心脏手术病人麻醉期间及术后镇静辅助用药，有可能通过减少全麻药的使用、降低应激反应程度、改善病人术后睡眠质量以及其可能的抗炎效应，降低心脏病人术后谵妄的发生率，从而改善患者的预后。

**3.试验目的**：

观察右美托咪定用于术中及术后心脏外科手术病人全麻辅助用药：（1）对术后谵妄的影响；（2）对术后认知功能恢复情况的影响；（3）对术后住院期间并发症的发生率和30天全因死亡率的影响。

**4.研究人群**

术前一天根据入选/排除标准对拟入组的患者进行筛选。如手术为周一，则筛选在之前的周五进行。

**4.1 入选标准**

年龄≥60岁、拟行心脏手术（CABG和/或瓣膜手术）的病人。

**4.2 排除标准**

凡具有下列情况之一者，不作为入选病例：

4.2.1.拒绝参加本研究；

4.2.2术前有精神分裂症、癫痫、帕金森症者；

4.2.3术前因严重痴呆、语言障碍或终末期疾病无法交流者；

4.2.4颅脑损伤或功能区神经外科手术史者；

4.2.5术前有病态窦房结综合征、严重窦性心动过缓(心率<50次/分)、II度以上房室传导阻滞且未植入起搏器者；

4.2.6严重肝功能异常（Child-Pugh C级)

4.2.7严重肾功能异常（术前接受肾脏替代治疗者）

4.2.8其他研究者认为不适宜入选的情况（需注明具体原因）。

**4.3脱落病例标准**

4.3.1病人自行退出（撤销同意书）；

4.3.2失访；

4.3.3研究者令其退出（出现严重不良事件）；

4.4.4失盲病例。

对于脱落病例，应在CRF表上详细记录原因并妥善保管备查，数据以最后一次评估的结局变量作为最终结果纳入统计分析。

**4.4 无效病例标准**

4.4.1研究药物未输注；

4.4.2无研究记录；

4.4.3因使用了试验中禁止使用的药物而无法进行终结局变量评价的病例。

对于无效病例，应在CRF表上详细记录原因且妥善保管备查。数据最终不纳入统计分析。

**4.5中止试验的标准**

研究进行中出现以下情况需中止试验：

4.5.1研究者发现与试验药物相关的严重安全性问题；

4.5.2方案有重大失误；

4.5.3申办方因经费或管理原因；

4.5.4行政主管部门撤销试验。

中止试验可是暂时的，也可是永久的。中止试验时，全部试验记录应予保留备查。

**5.研究总体设计**

本研究为两中心、前瞻性、随机、双盲、安慰剂对照研究，设有2个平行组。研究由北京大学第一医院发起，并在北京大学第一医院及中国医学科学院阜外心血管病医院麻醉科实施。

**6.样本量估算**

文献报道心脏手术术后的谵妄发生率在3%-47%[2]。根据我们以往的研究结果，我们估计对照组谵妄发生率为30%。一项荟萃分析显示[34]，使用右美托咪定作为心脏术后镇静使谵妄发生率降低约63%（RR 0.36, [95%CI : 0.21-0.64 ]），我们保守的假设本研究中右美托咪定组谵妄发生率为15%。取检验水准α＝0.05、检验效能1－β＝0.80推算，两组按1：1进行病例分配，每组各需要完成有效病例118例，共需要236例。考虑到20%的病例脱落，本研究预期入选病例284例。样本量计算由PASS 11软件（NCSS, LLC, Kaysville, Utah）完成

**7.随机、设盲和破盲**

**7.1随机：**

由与本试验执行数据管理统计分析无关的生物统计学家，在计算机上用SAS统计软件包，按试验组与对照组1:1的比例用中心分层随机化方法产生随机编码。试验药物（右美托咪定或生理盐水）均为无色透明液体，盛放于同样的3ml西林瓶（均由江苏恒瑞医药股份有限公司提供），由指定的药剂师（该药剂师不参加以后的研究）根据此随机数对药物进行编码，各个临床试验中心按分配的药物编号按病例入组次序依次使用。盲底由各研究中心保存直至全部试验结束。

**7.2盲法:**

7.2.1对研究人员施盲：指定一名研究协调员，负责保存和分发随机号码、准备药物以及研究人员之间的信息协调。指定一名主治医师负责麻醉实施和记录术中信息。由经过专业神经科医师培训的随访人员负责病人的随访和谵妄的诊断。以上研究人员在研究期间互相不知道其余研究人员的记录内容。

7.2.2对患者施盲：所有患者术中均使用统一外观的注射器、微量注射泵。

**7.3 破盲：**

当入组病例研究全部完成后，所有病例报告表经输入并核对无误，进行数据库锁定后进行揭盲，交统计分析人员进行统计分析。

**8.方案实施：**

**8.1 药品分发：**由研究协调员根据试验入组编号选择药物，即药物编码与入组患者的随机编号一致，并在CRF表中记录该编码。

**8.2 药品使用：**两组试验药物均用生理盐水稀释至50ml（右美托咪定浓度为4μg /ml）。在麻醉诱导前，右美托咪定组以[0.9×kg] ml/h速度静脉输注试验药物10min（相当于在10min内泵入0.6μg/kg右美托咪定），继以[0.1×kg] ml/h速度静脉输注至术毕（相当于干预组以0.4μg/kg/h速度输注右美托咪定），术后干预组病人继续以0.025×kg ml/h速度输注右美托咪定（相当于0.1 μg/kg/h）直至停止机械通气；对照组则以相同的速度输注生理盐水至停止机械通气。

**8.3麻醉管理：**

手术当日根据病人情况可给相应的术前药，但避免使用东莨菪碱和长托宁。患者入室，常规吸氧，监护五导联心电图、脉氧饱和度、有创动脉血压、BIS、中心静脉压和/或肺动脉压、鼻咽温、尿量。根据临床需要决定是否使用心排量监测设备或经食道超声。

麻醉诱导采用静脉麻醉诱导法，药物包括咪达唑仑、依托咪酯、舒芬太尼、丙泊酚等，麻醉维持采用舒芬太尼、丙泊酚、七氟烷吸入等，保证BIS在40-60之间。除试验药物外不使用右美托咪定。

术中根据血流动力学监测情况调整输液量和输液速度，维持血流动力学稳定。

术后患者常规入心外科监护室继续治疗。术后镇痛采用阿片类静脉注射/输注，或其他口服镇痛药；术后镇静采用以丙泊酚为基础的静脉镇静策略，维持RASS镇静评分在-2~1之间。必要时可静脉注射咪达唑仑。

**8.4 谵妄的处理：**

一旦诊断术后谵妄，应积极的处理。需从去除诱因、支持疗法、药物干预三方面入手。去除诱因的措施有：停用可能引起谵妄的药物、纠正水电解质平衡等。支持疗法包括：优质护理（再定向、认知刺激、早期活动、视听帮助）、恰当的热量和营养供应、纠正患者的睡眠-觉醒周期紊乱、家庭成员床旁陪同等。对于躁动型谵妄，可给予氟哌啶醇静脉注射（首次负荷量0.5-2mg，15-20min后可重复，直至症状控制，其后可根据需要每4-6小时静脉给予负荷量的半量），持续数日。用药期间应注意监测QT间期及有无锥体外系症状。必要时咨询精神科医师。

**9. 资料收集**

研究者在试验开始前统一经过数据收集及结局评估有关的培训。

**9.1术前：**

9.1.1采集人口学资料，包括姓名、性别、出生日期、文化程度、受教育年限、体重系数等；

9.1.2 术前诊断，合并疾病，最近一个月内的用药情况，既往手术史及麻醉史等；计算Charlson合并症指数分值；

9.1.3辅助检查：主要化验结果[血/尿/便常规、血生化全项（肌酐、肌钙蛋白）、凝血功能等]，心电图、心脏超声、胸片等；

9.1.4简明智力状态检查量表（MMSE）评分；

9.1.5医院焦虑/抑郁量表(Hospital Anxiety and Depression Scale, HAD)评分；

9.1.6巴氏指数（Barthel Index），评价日常生活活动能力；

9.1.7谵妄评估（CAM法）；

9.1.8血清样本：术前留取血液样本2管各2-3ml，置于专用采血管，并离心分离出血清样品，-80C深低温保存，分别进行血中s100β、Neutrophil Gelatinase Associated Lipocalin (NGAL)水平的测定。

**9.2术中**

记录手术时间、手术名称，麻醉时间、麻醉药物总用量，术中出血量、输血量、液体出入量及其他各种药物的使用情况；

**9.3术后**

9.3.1 血清样本：分别于术后6h及术后48h抽取血样2管各2-3ml，置于专用采血管，并离心分离出血清样品，-80C深低温保存。对术后6h血清样本进行Neutrophil Gelatinase Associated Lipocalin (NGAL)水平的测定、对术后48h血清样本进行s100β蛋白水平的测定；

9.3.2术后入ICU后24小时内的最差APACHE II评分；

9.3.3 谵妄评估：术后第1-5天每天1次，插管病人采用CAM-ICU、非插管病人采用CAM法对进行谵妄评估；

9.3.4疼痛评分：术后第1-5天每天上午8-10点用NRS法（0=完全无痛，10=最严重疼痛）评估患者静息状态及咳嗽时的疼痛评分；

9.3.5 主观睡眠质量评分：术后1-5天每天上午8-10采用NRS法（0=睡眠最好，10=睡眠最差）进行睡眠质量评分；

9.3.6 认知功能评估：术后第6天用MMSE法，在术后1月用TICS法进行认知功能评估；

9.3.7术后1-5天用药（原发疾病所需药物，镇静、镇痛、抗胆碱药以及糖皮质激素）情况及监护室出入量情况；

9.3.8术后机械通气时间、ICU停留时间、住院天数；。

9.3.9 术后30天内各系统并发症发生情况，如肺部并发症、急性肾损伤、心脑血管事件、感染等。

9.9.10术后30天的存活状态（电话随访）。

**10.结局指标**

**10.1 主要结局：**

术后前5天内谵妄的发生率。

**10.2 次要结局：**

10.2.1术后6天及30天的认知功能评估；

10.2.2术后30天内的非谵妄并发症的发生率；

10.2.3术后30天的全因死亡率。

**10.3其他结局：**

10.3.1术后1-5天的NRS疼痛评分；

10.3.2术后1-5天的主观睡眠质量NRS评分；

10.3.3术后机械通气时长；

10.3.4术后ICU停留时间及术后住院时间；

10.3.5术后30内的再入院率。

**11.不良事件及严重不良事件**

**11.1 不良事件**

11.1.1定义

无论与药物是否有关，凡是与研究中的任何医疗措施有关的、出现的非预期的、不利的医学事件均为不良事件。一个不良事件可以是任何非预测的和不适宜的体征（包括异常的实验室检查等）、症状或与使用产品相关的暂时性疾病。

11.1.2 本试验预期的不良事件

11.1.2.1心动过缓：心率<45次/分，或较术前基础水平下降超过30%并持续5min以上；

11.1.2.2低血压：收缩压<80mmHg，或较术前基础水平下降超过30%并持续5min以上；

11.1.2.3心动过速：心率>100次/分，或较术前基础水平增加超过30%并持续5min以上；

11.1.2.4高血压：收缩压>160mmHg，或较术前基础水平增加超过30%并持续5min以上；

11.1.2.5低氧合：脉氧饱和度<90%,或较基础水平下降超过5%。

11.1.3不良事件的处理

不良事件一旦出现，应根据临床医疗常规进行治疗，试验药物的速度可根据需要作短暂或长期下调，如麻醉医师或监护室医师认为有必要，可终止试验药物的输注，但需要CRF表详实记录停药时间及原因。

常见处理包括：心动过缓（静脉注射阿托品或输注异丙肾上腺素）；低血压（容量治疗及诸如麻黄碱、多巴胺、去甲肾上腺素等血管活性药的使用）；心动过速（静脉使用艾司洛尔、阿替洛尔或地尔硫卓）；高血压（静脉使用硝普钠、乌拉地尔、地尔硫卓等血管活性药）；低氧合（增加吸入氧浓度或调整呼吸机参数以及胸部物理治疗）。

11.1.4记录

任何发生的不良事件均应记录，包括种类、诊断时间、处理、持续时间、结局等。

不良事件随访至消失或治疗终结。

**11.2 严重不良事件**

11.2.1 定义

严重不良事件是指非预期的导致病人死亡、危及病人生命、永久性伤残或延长住院时间的其他严重情况的医学事件。

11.2.2严重不良事件的处理

如出现严重不良事件，除应积极治疗或抢救外，应在获知后24小时内以电话或传真形式通知临床研究负责人和伦理委员会。如发生与治疗相关的死亡事件，应立即停止临床试验，尽快上报伦理委员会，并详细记录和妥善保存有关资料。

所有严重不良事件都应当追踪，并及时记录不良事件的出现、类别、发生时间、程度、持续时间及转归。直到得到妥善解决或病情稳定。

**11.3 不良事件的监测期限**

从实验药物开始输注至术后24h。但不良事件一旦发生，应监测至其消失或治疗终结。

**12.数据管理**

12.1研究者根据受试者的原始观察记录，将数据及时、完整、正确的载入病例报告表；

12.2监查员监查试验的进行是否遵循试验方案。病例报告表经监查员签字后，及时送交临床试验数据保管员；

12.3病例报告表在按要求完成数据录入和核查后（一人录入，另一人核查），按编号的顺序归档保存。

12.4 数据监察由北京大学第一医院临床研究伦理委员会进行。

**13.统计学分析**

**13.1 一般原则：**

计量资料根据数据分布的形态采用均值（标准差）或中位数（四分位间距）表示；计数资料用例数（百分比）表示。所有的统计检验均采用双侧检验，P值小于或等于0.05将被认为所检验的差别有统计意义。

**13.2入组及完成情况：**

总结各中心入组及完成病例数，对两组总脱落率将采用卡方检验进行比较。

**13.3 一般信息与基线特征：**

对患者的人口学信息、用药史、合并其他疾病史等进行统计描述。对于实验组与对照组的基线数据比较，计量资料采用采用成组t检验／Wilcoxon秩和检验，计数资料采用卡方检验／Fisher精确检验。

**13.4. 疗效评价**

13.4.1主要结局评价：

对两组术后5日内各时间点的谵妄发生情况进行统计描述。两组谵妄发生率的比较采用卡方检验。二元logistic回归分析计算干预措施对术后谵妄发生率的危险度及其95%可信区间。

13.4.2 次要结局评价

13.4.2.1 术后6天及30天的认知功能评价：组间采用t检验或Wilcoxon秩和检验；

13.4.2.2 术后非谵妄并发症发生率：根据数据形态，组间采用卡方检验、连续校正的卡方检验或Fisher精确概率检验；

13.4.2.3 术后30天全因死亡率：采用Kaplan-Meier 生存分析，组间差异用对数秩检验；

13.4.3 其他结局评价：

13.4.3.1 术后1-5天疼痛及睡眠质量评分：组间采用Wilcoxon秩和检验；

13.4.3.2 术后机械通气时间、ICU停留时间、术后住院时间; 根据数据形态，采用独立样本t检验或Wilcoxon秩和检验；

13.4.3.3 术后30天再入院率：根据数据形态，组间采用卡方检验、连续校正的卡方检验或Fisher精确概率检验；

**14．质量控制**

14.1研究开始前向研究人员和医务人员详细解释研究方案。研究期间必须必须严格遵循研究方案；

14.2研究人员应完整、详细、准确的填写病例报告表。临床试验中所有观察到的结果和异常发现，均应及时加以认真核实、记录，保证数据的可靠性；

14.3试验中各种检查项目所使用的各种仪器设备，均应有严格的质量标准，并确保正常工作；

14.4统计分析由专业生物统计学的人员完成；

14.5所有结论应基于原始数据得出。

**15伦理学要求**

15.1遵循赫尔辛基宣言和中国有关临床试验管理规范进行临床试验。在试验开始之前，由临床研究伦理委员会批准试验方案后方可实施临床试验；

15.2术前研究人员到病房向符合筛选条件的患者或/及其委托人充分说明本研究的研究目的、方法及其可能带来的益处和危害，并详细解答患者及家属的疑问，给予其充分的考虑时间，在完全自愿的前提下请患者或/及其委托人签署知情同意书。对术前已明确存在痴呆等神经精神疾病或无自主行为能力的患者，应具备委托人签字。知情同意书作为研究文件保存；

15.3所有与研究相关的个人信息均属保密资料，不允许拷贝。除参与本临床研究的研究人员、伦理委员会、研究单位相关管理部门外，不得向其他任何人、任何单位透露。

**16资料保存**

研究者、申办者应当按照GCP要求对临床试验的文件和资料进行妥善保存。

**17预期进度**

17.1临床试验观察期：12-24个月。主要包括入选患者的筛选、入选、治疗及随访；

17.2数据处理和资料总结期：6-12个月。主要对临床试验所得资料进行统计学处理、资料的总结。

**18．****References:**

1. Association AP: **Diagnostic And Statistical Manual Of Mental Disorders. 4th Ed (DSM-IV).**; 1994.

2. Groen JA, Banayan D, Gupta S, Xu S, Bhalerao S: **Treatment of delirium following cardiac surgery**. *J Card Surg* 2012, **27**(5):589-593.

3. Koster S, Hensens AG, Schuurmans MJ, van der Palen J: **Consequences of delirium after cardiac operations**. *ANN THORAC SURG* 2012, **93**(3):705-711.

4. Jodati A, Safaie N, Raoofi M, Ghorbani L, Ranjbar F, Noorazar G, Mosharkesh M: **Prevalence and risk factors of postoperative delirium in patients undergoing open heart surgery in northwest of iran**. *J Cardiovasc Thorac Res* 2013, **5**(3):97-99.

5. Brown CH: **Delirium in the cardiac surgical ICU**. *Curr Opin Anaesthesiol* 2014, **27**(2):117-122.

6. Leslie DL, Inouye SK: **The importance of delirium: economic and societal costs**. *J AM GERIATR SOC* 2011, **59 Suppl 2**:S241-S243.

7. Milbrandt EB, Deppen S, Harrison PL, Shintani AK, Speroff T, Stiles RA, Truman B, Bernard GR, Dittus RS, Ely EW: **Costs associated with delirium in mechanically ventilated patients**. *CRIT CARE MED* 2004, **32**(4):955-962.

8. Koster S, Hensens AG, van der Palen J: **The long-term cognitive and functional outcomes of postoperative delirium after cardiac surgery**. *ANN THORAC SURG* 2009, **87**(5):1469-1474.

9. Loponen P, Luther M, Wistbacka JO, Nissinen J, Sintonen H, Huhtala H, Tarkka MR: **Postoperative delirium and health related quality of life after coronary artery bypass grafting**. *SCAND CARDIOVASC J* 2008, **42**(5):337-344.

10. Martin BJ, Buth KJ, Arora RC, Baskett RJ: **Delirium: a cause for concern beyond the immediate postoperative period**. *ANN THORAC SURG* 2012, **93**(4):1114-1120.

11. Gottesman RF, Grega MA, Bailey MM, Pham LD, Zeger SL, Baumgartner WA, Selnes OA, McKhann GM: **Delirium after coronary artery bypass graft surgery and late mortality**. *ANN NEUROL* 2010, **67**(3):338-344.

12. Chan MT, Cheng BC, Lee TM, Gin T: **BIS-guided anesthesia decreases postoperative delirium and cognitive decline**. *J Neurosurg Anesthesiol* 2013, **25**(1):33-42.

13. Radtke FM, Franck M, Lendner J, Kruger S, Wernecke KD, Spies CD: **Monitoring depth of anaesthesia in a randomized trial decreases the rate of postoperative delirium but not postoperative cognitive dysfunction**. *Br J Anaesth* 2013, **110 Suppl 1**:i98-i105.

14. Plaschke K, Fichtenkamm P, Schramm C, Hauth S, Martin E, Verch M, Karck M, Kopitz J: **Early postoperative delirium after open-heart cardiac surgery is associated with decreased bispectral EEG and increased cortisol and interleukin-6**. *Intensive Care Med* 2010, **36**(12):2081-2089.

15. Cerejeira J, Batista P, Nogueira V, Vaz-Serra A, Mukaetova-Ladinska EB: **The stress response to surgery and postoperative delirium: evidence of hypothalamic-pituitary-adrenal axis hyperresponsiveness and decreased suppression of the GH/IGF-1 Axis**. *J Geriatr Psychiatry Neurol* 2013, **26**(3):185-194.

16. Tan MC, Felde A, Kuskowski M, Ward H, Kelly RF, Adabag AS, Dysken M: **Incidence and predictors of post-cardiotomy delirium**. *Am J Geriatr Psychiatry* 2008, **16**(7):575-583.

17. Kosar CM, Tabloski PA, Travison TG, Jones RN, Schmitt EM, Puelle MR, Inloes JB, Saczynski JS, Marcantonio ER, Meagher D *et al*: **EFFECT OF PREOPERATIVE PAIN AND DEPRESSIVE SYMPTOMS ON THE DEVELOPMENT OF POSTOPERATIVE DELIRIUM**. *Lancet Psychiatry* 2014, **1**(6):431-436.

18. Leung JM, Sands LP, Lim E, Tsai TL, Kinjo S: **Does preoperative risk for delirium moderate the effects of postoperative pain and opiate use on postoperative delirium?** *Am J Geriatr Psychiatry* 2013, **21**(10):946-956.

19. Burkhart CS, Dell-Kuster S, Gamberini M, Moeckli A, Grapow M, Filipovic M, Seeberger MD, Monsch AU, Strebel SP, Steiner LA: **Modifiable and nonmodifiable risk factors for postoperative delirium after cardiac surgery with cardiopulmonary bypass**. *J Cardiothorac Vasc Anesth* 2010, **24**(4):555-559.

20. Norkienė I, Ringaitienė D, Kuzminskaitė V, Šipylaitė J: **Incidence and Risk Factors of Early Delirium after Cardiac Surgery**. *BIOMED RES INT* 2013, **2013**:1-5.

21. Smulter N, Lingehall HC, Gustafson Y, Olofsson B, Engstrom KG: **Delirium after cardiac surgery: incidence and risk factors**. *INTERACT CARDIOV TH* 2013, **17**(5):790-796.

22. Fitzgerald JM, Adamis D, Trzepacz PT, O'Regan N, Timmons S, Dunne C, Meagher DJ: **Delirium: a disturbance of circadian integrity?** *MED HYPOTHESES* 2013, **81**(4):568-576.

23. Trompeo AC, Vidi Y, Locane MD, Braghiroli A, Mascia L, Bosma K, Ranieri VM: **Sleep disturbances in the critically ill patients: role of delirium and sedative agents**. *MINERVA ANESTESIOL* 2011, **77**(6):604-612.

24. Gerresheim G, Schwemmer U: **[Dexmedetomidine]**. *ANAESTHESIST* 2013, **62**(8):661-674.

25. Harsoor SS, Rani DD, Lathashree S, Nethra SS, Sudheesh K: **Effect of intraoperative Dexmedetomidine infusion on Sevoflurane requirement and blood glucose levels during entropy-guided general anesthesia**. *J Anaesthesiol Clin Pharmacol* 2014, **30**(1):25-30.

26. Naguib AN, Tobias JD, Hall MW, Cismowski MJ, Miao Y, Barry N, Preston T, Galantowicz M, Hoffman TM: **The role of different anesthetic techniques in altering the stress response during cardiac surgery in children: a prospective, double-blinded, and randomized study**. *Pediatr Crit Care Med* 2013, **14**(5):481-490.

27. Wang Y, Xu X, Liu H, Ji F: **Effects of dexmedetomidine on patients undergoing radical gastrectomy**. *J SURG RES* 2015, **194**(1):147-153.

28. Abd AN, Chue MC, Yong CY, Hassan Y, Awaisu A, Hassan J, Kamarulzaman MH: **Efficacy and safety of dexmedetomidine versus morphine in post-operative cardiac surgery patients**. *Int J Clin Pharm* 2011, **33**(2):150-154.

29. Barletta JF, Miedema SL, Wiseman D, Heiser JC, McAllen KJ: **Impact of dexmedetomidine on analgesic requirements in patients after cardiac surgery in a fast-track recovery room setting**. *PHARMACOTHERAPY* 2009, **29**(12):1427-1432.

30. Maldonado JR, Wysong A, van der Starre PJ, Block T, Miller C, Reitz BA: **Dexmedetomidine and the reduction of postoperative delirium after cardiac surgery**. *PSYCHOSOMATICS* 2009, **50**(3):206-217.

31. Ji F, Li Z, Young N, Moore P, Liu H: **Perioperative dexmedetomidine improves mortality in patients undergoing coronary artery bypass surgery**. *J Cardiothorac Vasc Anesth* 2014, **28**(2):267-273.

32. Curtis JA, Hollinger MK, Jain HB: **Propofol-based versus dexmedetomidine-based sedation in cardiac surgery patients**. *J Cardiothorac Vasc Anesth* 2013, **27**(6):1289-1294.

33. Dasta JF, Jacobi J, Sesti AM, McLaughlin TP: **Addition of dexmedetomidine to standard sedation regimens after cardiac surgery: an outcomes analysis**. *PHARMACOTHERAPY* 2006, **26**(6):798-805.

34. Lin YY, He B, Chen J, Wang ZN: **Can dexmedetomidine be a safe and efficacious sedative agent in post-cardiac surgery patients? a meta-analysis**. *CRIT CARE* 2012, **16**(5):R169.

**Impact of dexmedetomidine on the Incidence of delirium in elderly patients after cardiac surgery: A multicenter, randomized, double-blind, placebo-controlled trial**

**Study protocol**

**Study Drug**: dexmedetomidine hydrochloride for injection

**Principal Investigator**: Dong-Xin Wang, MD, PhD

**Study Design**: Dong-Xin Wang, MD, PhD; Xue Li, MD; Li-Huan Li, MD, PhD and Daqing Ma, MD, PhD, FRCA

**Name of Institute**: Department of Anesthesiology and Critical Care Medicine (Dong-Xin Wang, MD, PhD and Xue Li, MD), Peking University First Hospital, Beijing 100034, China; Department of Anesthesiology (Li-Huan Li, MD, PhD), Fuwai Hospital, National Center for Cardiovascular Diseases, Chinese Academy of Medical Sciences and Peking Union Medical College, Beijing 100037, China; and Section of Anaesthetics, Pain Management and Intensive Care (Daqing Ma, MD, PhD, FRCA), Department of Surgery and Cancer, Imperial College London, Chelsea and Westminster Hospital, London, UK.

**Version: 2.0**

**Date of version: September 10, 2014**

**1. Abstract**

**1.1. Background**

Retrospective studies showed that for patients undergoing cardiac surgery, use of dexmedetomidine during the perioperative period was associated with decreased risk of delirium. However, evidences from prospective randomized controlled trials are still lacking.

**1.2. Objectives**

To investigate the hypothesis that dexmedetomidine, when used as an adjunctive drug during both general anesthesia and postoperative sedation, would decrease the incidence of delirium in elderly patients after cardiac surgery.

**1.3. Study design**

This is a two-center, perspective, randomized, double-blind, placebo-controlled trial.

**1.4. Setting**

Two tertiary hospitals (Peking University First Hospital and Beijing Fuwai Hospital) in Beijing.

**1.5. Patients**

Two hundred and eighty four elderly patients (age ≥ 60 years) who are scheduled to undergo coronary artery bypass graft and/or valve surgery.

**1.6. Intervention**

1.6.1. For patients in the dexmedetomidine (DEX) group, a loading dose dexmedetomidine will be administered 10 minutes before anesthesia (0.6 μg/kg in 10 minutes), followed by a continuous infusion at a rate of 0.4 μg/kg/h until the end of surgery. After surgery, the infusion rate will be decreased to 0.1 μg/kg/h until the end of mechanical ventilation;

1.6.2. For patients in the control (CTRL) group, normal saline will be administered in the same rate and volume as that in the DEX group.

**1.7. Primary outcome**

The incidence of delirium during the first 5 days after surgery.

**1.8. Predicted duration of the study**

2-3 years.

**1.9. Trial registration**

The study is registered with [ClinicalTrials.gov](http://www.clinicaltrials.gov/), number NCT02267538.

**2. Background**

Delirium is an acute mental syndrome characterized by disturbance of consciousness, attention, cognition and perception that develops over a short period of time (usually hours to days) and tending to fluctuate during the course of the day [1]. It is a common complication in elderly patients after cardiac surgery, with reported incidence ranging from 3% to 47% [2]. The occurrence of postoperative delirium (POD) is associated with worse outcomes, including prolonged length of stay in the ICU and hospital [3, 4], increased morbidity [5], and high medical care costs [6, 7]. Moreover, prolonged delirium is associated with worse long-term outcomes, including compromised cognitive function [8], lowered life quality [9], and elevated mortality [10, 11].

The causes of POD are multifactorial and may include deep anesthesia [12, 13], severe surgical stress [14, 15], intense postoperative pain [16, 17], high dose opioid analgesia [18, 19], prolonged mechanical ventilation [20, 21], and sleep disturbances after surgery [22, 23]. T[heoretically](app:ds:theoretically), measures that decreases anesthetic consumption (but maintains adequate anesthesia depth), [alleviates surgical](app:ds:alleviate) stress response, improves postoperative analgesia, reduces opioid and sedative requirement, and promotes sleep quality will be able to decrease the incidence of POD.

Dexmedetomidine is a highly selective α2–adrenoreceptor agonist that provides sympatholytic, sedative, and analgesic effects. It has been increasingly used during clinical anesthesia and postoperative sedation [24]. Studies showed that, when used as an adjunctive drug during general anesthesia, dexmedetomidine decreased the consumption of anesthetics and opioids and suppressed the severity of stress response [25-27]; when used during the postoperative period, dexmedetomidine decreased the requirement of other sedatives and analgesics, improved the quality of analgesia and sleep, and reduced the incidence of delirium [28-31]. Retrospective studies showed that for patients undergoing cardiac surgery, use of dexmedetomidine during the perioperative period was associated with decreased risk of delirium, non-delirium complications and one-year mortality after surgery [31-33]. However, clinical evidences from prospective randomized controlled trials are still lacking.

We hypothesize that, for patients undergoing cardiac surgery, use of dexmedetomidine as an adjuvant during general anesthesia and postoperative sedation may reduce the incidence of postoperative delirium, possibly by decreasing the consumption of general anesthetics, relieving the severity of stress response, improving postoperative sleep, and suppressing the degree of inflammation.

**3. Purpose of the study**

To investigate the effects of dexmedetomidine, when used as an adjunctive drug during general anesthesia and postoperative sedation, on the incidence of delirium, the recovery of cognitive function, the occurrence of complications, and the 30-day mortality in elderly patients after cardiac surgery.

**4. Recruitment of participants**

Potential participants will be screened the day before surgery or, for those who will undergo surgery on Monday, on Friday before surgery.

**4.1. Inclusion criteria**

4.1.1. Elderly patients (age above 60 years);

4.1.2. Scheduled to undergo coronary artery bypass graft and/or valve surgery.

**4.2. Exclusion criteria**

Patients who meet any of the following criteria will be excluded:

4.2.1. Refuse to participate;

4.2.2. Previous history of schizophrenia, epilepsy or Parkinson disease;

4.2.3. Unable to complete preoperative assessment due to severe dementia, language barrier or end-stage disease;

4.2.4. Previous history of functional neurosurgery or brain injury;

4.2.5. Preoperative sick sinus syndrome, severe bradycardia (HR <50 bpm), second-degree or above atrioventricular block without pacemaker;

4.2.6. Severe hepatic insufficiency (Child-Pugh grades C);

4.2.7. Severe renal insufficiency (requirement of renal replacement therapy);

4.2.8. Any other conditions that are considered unsuitable for study participation.

**4.3. Criteria of drop out**

4.3.1. Withdrawn consents by the participants themselves;

4.3.2. Loss to follow-up;

4.3.3. Ordered to exit by the investigators or attending physicians (occurrence of severe adverse events or severe complications);

4.3.4. Cases of unmasked blindness.

For drop out cases, the detailed reasons will be recorded and the primary therapeutic effects recorded in the last time will be regarded as the final results. The Case Report Forms (CRFs) of these cases will be preserved for future reference.

**4.4. Criteria of rejection**

Enrolled cases who meet any of the following criteria will be excluded from further per protocol analysis.

4.4.1. Study drug is not administered;

4.4.2. No study record;

4.4.3. Unable to evaluate the effectiveness and safety because of the use of any prohibited drugs.

For rejected cases, the detailed reasons will be recorded and CRFs will be preserved for reference. The results of these cases will be excluded for further analysis of therapeutic effects.

**4.5. Criteria of study interruption**

Study will be interrupted in the following situations:

4.5.1. Severe safety problem occurred during the study;

4.5.2. Serious mistake found in the protocol;

4.5.3. Fund or management problem of the investigators;

4.5.4. Study cancelled by the administrative authority.

Study interruption may transient or permanent. All recorded CRFs will be preserved for reference in case of study interruption.

**5. Study Design**

5.1. This is a double-center, perspective, randomized, double-blind, placebo controlled clinical trial with two parallel arms;

5.2. It is coordinated by the Department of Anesthesiology and Critical Care Medicine of Peking University First Hospital;

5.3. The study will be conducted in Peking University First Hospital and Beijing Fuwai Hospital.

**6. Sample Size Estimation**

Studies showed that the incidence of postoperative delirium in cardiac surgery patients varied from 3% to 47% [2]. According to the results of our previous study, we assume that the incidence of delirium is 30% in the control group. A recently meta-analysis showed that, dexmedetomidine can reduce the incidence of delirium by 63% (RR 0.36, 95% CI 0.21-0.64) when used as postoperative sedation [34] . We conservatively assume that delirium will be reduced by 50% in the dexmedetomidine group in this study, i.e., 15%. With significance and power set at 0.05 (two-sided) and 80% respectively, the sample size required to detect this difference is 236 patients. Taking into account a 20% loss-to-follow up rate, we plan to enroll 284 patients. Sample size calculation is performed with the PASS 11 software (NCSS, LLC, Kaysville, Utah).

**7. Randomization, blinding and unmask of blinding**

**7.1. Randomization**

7.1.1. A biostatistician who does not participate in data management and statistical analysis will generate random numbers in a 1:1 ratio using the SAS statistical package version 9.3 (SAS Institute, Cary, NC, USA). Randomization will be stratified by centers;

7.1.2. Study drugs (dexmedetomidine chloride 200 μg/2 mL and normal saline 2 mL) will be provided as clear aqueous solutions in the same 3 mL bottles. A pharmacist who does not participate in the rest of the study will encode the study drugs according to the randomization sequence;

7.1.3. The randomization results will be sealed in sequentially numbered letters and stored at the site of investigation until the end of the study.

**7.2. Blinding**

7.2.1. A study coordinator will be assigned to distribute the study drugs according to the sequence of recruited patients, and to coordinate between investigators;

7.2.2. For each recruited patient, an anesthesiologist will be assigned for anesthesia management and intraoperative data collection;

7.2.3. For each recruited patient, an investigator/nurse will be assigned for study drug administration;

7.2.4. Postoperative follow-up and delirium assessment will be performed by investigators who have been trained prior to the study;

7.2.5. The anesthesiologist and the investigator do not know the results they collected each other;

7.2.6. All syringes and injection pumps used for study drug administration were same in external appearance.

**7.3.** **Unmask of blinding**

After each trial patient’s study is completed and all data are inputted into the database and checked without mistakes, the database will be locked up and the blindness will be unmasked. The database will then be sent to a biostatistician for statistical analysis.

**8. Intervention and anesthesia management**

**8.1. Distribution of study drugs**

8.1.1. Each of the recruited patients will be assigned a number according to the sequence of recruitment;

8.1.2. A study coordinator will distribute the study drugs according to the number of patient recruitment, i.e., the number of recruitment will be the same of the number of study drugs;

8.1.3. The number of recruitment/study drugs will be recorded in the CRF by the investigator.

**8.2. Administration of study drugs**

8.2.1. Study drugs will be diluted with normal saline to 50ml (for dexmedetomidine chloride, the final concentration is 4 μg/mL);

8.2.2. Study drug infusion will begin at least 10 minutes before anesthesia induction at a rate of [0.9×kg] ml/h for10 minutes (i.e., 0.6 μg/kg in 10 minutes for dexmedetomidine), then at a rate of [0.1×kg] ml/h (i.e., 0.4 μg/kg/h for dexmedetomidine) until the end of surgery. After surgery, study drug will be infused at a rate of [0.025×kg] ml/h (i.e., 0.1 μg/kg/h for dexmedetomidine) until the end of mechanical ventilation.

**8.3. Anesthesia management**

8.3.1. Premedication may be administered before anesthesia according to patients’ condition;

8.3.2. Intraoperative monitoring includes electrocardiogram, pulse [oxygen saturation](app:ds:oxygen saturation), invasive arterial pressure, central venous pressure, nasopharyngeal temperature, bispectral index (BIS), and urine output. Other cardiac monitoring (thermodilution cardiac output, transesophageal echocardiography, etc.) will be performed when necessary;

8.3.3. Anesthesia will be induced with midazolam, etomidate, sufentanil, and propofol; and will be maintained with sufentanil infusion, combined with propofol infusion and/or sevoflurane inhalation. BIS will be maintained between 40 and 60;

8.3.4. During surgery, hemodynamics will be managed by adjusting fluid infusion and using inotropic and/or vasoactive drugs;

8.3.5. Patients will be transferred to the intensive care unit (ICU) after surgery;

8.3.6. Patient-controlled intravenous analgesia (with opioids) will be provided whenever possible. For patients who require additional analgesia, opioids or other analgesics will be administered either intravenously or orally. For patients who require sedation (for mechanical ventilation), propofol intravenous infusion was the first choice, the target Richmond Agitation Sedation Scale (RASS) is between -2 and +1. Midazolam will be administered when necessary.

**8.4. Prohibited medication**

8.4.1. Penehyclidine and scopolamine are prohibited;

8.4.2. Open-labled dexmedetomidine is not allowed.

**8.5. Management of delirium**

8.5.1. Precipitating factors (usually multiple precipitating factors exist) should be identified and corrected or managed;

8.5.2. Supportive care including reorientation, cognitive stimulation, early mobilization, hearing or vision aids, sleep-promotion, and nutrition supply should be administered for all delirious patients. Family members should be included in the supportive care of delirious patients;

8.5.3. Pharmacological treatment is only reserved for patients with severe agitation (RASS of +3 or higher). Haloperidol (0.5 to 2 mg) will be injected intravenously, repeated when necessary every 15 to 20 minutes until control of agitation. For maintenance treatment, half of the loading dose will be administered every 4 to 6 hours. The treatment can last for several days. QT internal and extrapyramidal symptoms will be monitored during haloperidol therapy. Psychiatrists will be consulted when necessary.

**9. Data collection**

Investigators performing data collection and assessment will be trained and qualified before the study.

**9.1. Preoperative data**

9.1.1. Demographic data, including gender, height, weight, date of birth, education, etc., will be collected;

9.1.2. Preoperative diagnosis, comorbidity (Charlson Comorbidity Index), history of medication (especially within one month), history of previous anesthesia and surgery will be collected;

9.1.3. Results of primary examination, such as electrocardiogram, echocardiogram, hematological and biochemical test results, will be collected;

9.1.4. Cognitive function will be assessed with Mini-Mental State Examination (MMSE);

9.1.5. Anxiety and depression will be assessed with Hospital Anxiety and Depression Scale;

9.1.6. Activity of daily living will be assessed with Barthel Index;

9.1.7. Delirium will be assessed with Confusion Assessment Method (CAM);

9.1.8. Blood sample will be collected. Serum will be separated by centrifugation and stored at a profound hypothermic refrigerator of -80°C for the measurement of neutrophil gelatinase associated lipocalin (NGAL) and S100B protein.

**9.2. Intraoperative data**

9.2.1. Duration of anesthesia, type and dose of drugs used during anesthesia;

9.2.2. Fluid balance and transfusion of blood products;

9.2.3. Type and duration of surgery.

**9.3. Postoperative data**

9.3.1. Blood samples will be collected at 6 and 48 hours after surgery. Serum will be separated by centrifugation and stored at a profound hypothermic refrigerator of -80°C for the measurement of NGAL (6 hr) and S100β protein (48 hr);

9.3.2. The worst Acute Physiology and Chronic Health Evaluation II (APACHE II) score during the first 24 hours after surgery will be recorded;

9.3.3. Delirium will be assessed daily during postoperative days 1 to 5 (8:00-10:00 am). Patients with endotracheal intubation will be assessed with the Confusion Assessment Method for the ICU (CAM-ICU); patients without endotracheal intubation will be assessed with CAM;

9.3.4. Pain score will be assessed daily during postoperative days 1 to 5 (8:00-10:00 am) with the Numeric Rating Scale (NRS, a 11-point scale where 0 indicates no pain and 10 indicates the worst pain);

9.3.5 Subjective sleep quality will be assessed daily during postoperative days 1 to 5 (8:00-10:00 am) with the NRS (a 11-point scale where 0 indicates the best possible sleep and 10 indicates the worst possible sleep);

9.3.6. Cognitive function will be assessed with MMSE at postoperative day 6, with Telephone Interview for Cognitive Status-modified (TICS-m) at postoperative day 30;

9.3.7. Postoperative medications (sedatives, analgesics, anticholinergics, glucocorticoids, etc.) during postoperative days 1-5;

9.3.8. Duration of mechanical ventilation, lengths of stay in the ICU and hospital after surgery;

9.3.9. Occurrence of non-delirium complications (pulmonary complications, acute kidney injury [diagnosed according to KDIGO-AKI criteria], cardiac events, cerebrovascular events, infections, etc.) during the first 30 days after surgery;

9.3.10. All-cause 30-day mortality after surgery.

**10. Outcomes**

**10.1. Primary outcome**

Incidence of delirium within the first 5 days after surgery.

**10.2. Secondary outcomes**

10.2.1. Cognitive function at postoperative days 6 and 30;

10.2.2. Incidence of non-delirium complications after surgery (until 30 days after surgery);

10.2.3. All-cause 30-day mortality after surgery.

**10.3. Additional outcomes**

10.3.1. NRS pain score at postoperative days 1-5;

10.3.2. NRS score of subjective sleep quality at postoperative days 1-5;

10.3.3. Duration of mechanical ventilation after surgery;

10.3.4. Length of stay in ICU and length of stay in hospital after surgery;

10.3.5. Rate of re-hospitalization within 30 days after surgery.

**11. Adverse events and severe adverse events**

**11.1.** **Adverse events**

**11.1.1. Definition**

An adverse event indicates any unpredictable, unfavorable medical event that is associated with any medical intervention and occurs during the study period. It can be related to the study drug administration or otherwise. It can manifest as any uncomfortable signs (including abnormal laboratory findings), symptoms or transient morbidity;

**11.1.2. Predicted adverse events in this study**

11.1.2.1. Bradycardia: heart rate < 45 beat per minute or a decrease of more than 30% from baseline (average value in the ward), and lasting for at least 5 minutes;

11.1.2.2. Hypotension: systolic blood pressure < 80 mmHg or a decrease of more than 30% from baseline, and lasting for at least 5 minutes;

11.1.2.3. Tachycardia: heart rate > 100 beats per minute or an increase of more than 30% from baseline, and lasting for at least 5 minutes;

11.1.2.4. Hypertension: systolic blood pressure > 160 mmHg or an increase of more than 30% from baseline, and lasting for at least 5 minutes;

11.1.2.5 Desaturation: pulse oxygen saturation < 90% or a decreased of more than 5% (absolute value) from baseline.

**11.1.3. Management**

11.1.3.1. Therapy will be provided according to routine clinical practice;

11.1.3.2. The study drug infusion rate can be decreased temporarily or permanently, or the study drug infusion can be stopped temporarily or permanently if considered necessarily by the attending anesthesiologists. The time and reasons of study drug interruption will be recorded in the CRFs;

11.1.3.3. Bradycardia: administration of medication (atropine iv bolus and/or isoprenaline iv infusion only);

11.1.3.4. Hypotension: intravenous fluid bolus, administration of medication (ephedrine/phenylephrine iv bolus, dopamine/dobutamine/epinephrine/norepinephrine, iv infusion);

11.1.3.5. Tachycardia: administration of medication (esmolol/diltiazem/atenolol iv infusion);

11.1.3.6. Hypertension: administration of medication (sodium nitroprusside/ditiazem/ urapidil/nicardipine iv bolus/infusion);

11.1.3.7. Desaturation: administration of oxygen (for patients without endotracheal intubation), adjustment of ventilator setting (for patients with endotracheal intubation), and/or physical therapy.

**11.1.4. Record**

11.1.4.1. Any adverse event should be documented, including occurrence, type, time of diagnosis, management, duration of persistence, and sequelae;

11.1.4.2. Any adverse event should be followed up until it is completely resolved or therapy termination.

**11.2. Severe adverse events**

**11.2.1. Definition**

A severe adverse event indicates any unpredictable medical events that lead to death, threat of life, prolonged length of hospital stay, persistent disability or dysfunction, or other severe event.

**11.2.2. Management**

In case of any severe adverse events, the study drug infusion will be stopped and treatment will be initiated immediately.

**11.2.3. Record and report**

11.2.3.1. In case of any severe adverse event, apart from active treatment and record as above, the principal investigator and the Ethics Committee will be informed within 24 hours in written report;

11.2.3.2. In case of study drug related death, immediately stop the clinical trial, report the event to the Ethics Committee as soon as possible, record in detail and carefully preserve the related documents;

11.2.3.3. Any severe adverse event must be followed up until it is completely resolved or when therapy is ended.

**11.3. Monitoring and follow up**

11.3.1. Adverse events and severe adverse events will be monitored from start of study drug infusion until 24 hours after surgery;

11.3.2. If an adverse event or a severe adverse event occurs, it will be followed up until complete resolution or therapy termination.

**12. Data management**

12.1. Investigators should promptly, completely, and correctly record data in the CRF according to original observation;

12.2. Supervisors will monitor if the study is carried out according to the protocol. The completed CRFs, after signed by the supervisor, will be sent to an investigator who is responsible for data management;

12.3. Data input will be performed by one investigator and checked out by another independent researcher. CRFs will be stored in sequence order;

12.4. Data management will be inspected by the Clinical Research Ethics Committee of Peking University First Hospital.

**13. Statistical analysis**

**13.1. General principles**

13.1.1. Numeric variables will be presented as mean (standard deviation) or median (minimum, maximum; or interquartile range). Categorical variables will be presented as number of cases (percentage);

13.1.2. Two-tailed tests will be used in all statistical analysis, and p values of less than 0.05 will be considered to be of statistical significance (unless otherwise indicated).

**13.2. Patient recruitment and drop-out situation**

The status of patient recruitment and drop-out will be summarized and listed. Comparison of the overall drop-out rate between the two groups will be performed with chi-square test.

**13.3. Demographics and baseline characteristics**

13.3.1. Demographic information and baseline characteristics (such as previous history of comorbidity and medication) will be presented;

13.3.2. Comparison of baseline numeric variables (such as age, BMI etc.) between groups will be performed with independent sample t-test or Wilcoxon rank sum test. Comparison of categorical variables (such as gender, presence of a comorbidity etc.) between groups will be performed with chi-square test or Fisher exact test.

**13.4. Effectiveness evaluation**

**13.4.1. Evaluation of primary outcome**

The incidence of delirium within the first 5 days after surgery will be calculated. Comparison between groups will be performed with chi-square test. The logistic regression analysis will be performed to evaluate risk for postoperative delirium with odds ratio (OR) and 95% CI as measures of association.

**13.4.2. Evaluation of secondary outcomes**

13.4.2.1. Cognitive function at postoperative days 6 and 30: comparison between groups will be performed with independent sample t-test or Wilcoxon rank sum test;

13.4.2.2. Incidence of postoperative non-delirium complications: comparison between groups will be performed with chi-square test or Fisher’s exact test;

13.4.2.3. All cause30-day mortality: comparison between groups will be performed with Kaplan-Meier survival analysis and the difference between groups will be tested by the log-rank test.

**13.5. Evaluation of additional outcomes**

10.3.1. NRS pain score at postoperative days 1-5: comparison between groups will be performed with Wilcoxon rank sum test;

10.3.2. NRS score of subjective sleep quality at postoperative days 1-5: comparison between groups will be performed with Wilcoxon rank sum test;

10.3.3. Duration of mechanical ventilation after surgery: comparison between groups will be performed with independent sample t-test or Wilcoxon rank sum test;

10.3.4. Length of stay in ICU and length of stay in hospital after surgery: comparison between groups will be performed with independent sample t-test or Wilcoxon rank sum test;

10.3.5. Rate of re-hospitalization within 30 days after surgery: comparison between groups will be performed with chi-square test.

**14. Quality control**

14.1. Trial protocol will be thoroughly explained to all investigators/care givers before the start of the trial. The trial protocol must be strictly adhered throughout the trial period;

14.2. All expected and unexpected findings will be documented promptly and correctly in order to guarantee the reliability of the values;

14.3. The monitors and instruments that are used during the study period will be checked and corrected regularly in order to guarantee their normal work;

14.4. Data analysis will be performed by the biostatisticians and investigators;

14.5. Any conclusions must be derived from the original data.

**15. Ethical issues**

15.1. Helsinki declaration and Chinese guidelines of Good Clinical Practice will be strictly followed. The study protocol must be approved by the Ethics Committee before the study can be started;

15.2. For every potential participant, investigators have the responsibilities to fully explain the study purpose, procedures, as well as possible risks in a written informed manner. They must let every potential participant know that he/she has the right to withdraw his/her consent at any time during the study period. Every potential participant must be given a written informed consent. Every participant or the authorized surrogate of the patient must sign the consent before they can be enrolled in the study. Written informed consents will be kept as a part of the clinical trial documents;

15.3. Personal information of all participants will be kept secret. Results of the study will be published in the form of theses, but personal information (including name, age, etc.) will be kept secret.

**16. Preservation of documents**

Investigators will carefully preserve all documents and data of the clinical trial according to the Good Clinical Practice requirement.

**17. Anticipated schedule**

17.1. Recruitment of participants: 12-24 months;

17.2. Data analysis and draft manuscript: 6-12 months.

**18. References:**

1. Association AP: **Diagnostic And Statistical Manual Of Mental Disorders. 4th Ed (DSM-IV)**. Washington, DC; 1994.

2. Groen JA, Banayan D, Gupta S, Xu S, Bhalerao S: **Treatment of delirium following cardiac surgery**. *J Card Surg* 2012, **27**(5):589-593.

3. Koster S, Hensens AG, Schuurmans MJ, van der Palen J: **Consequences of delirium after cardiac operations**. *ANN THORAC SURG* 2012, **93**(3):705-711.

4. Jodati A, Safaie N, Raoofi M, Ghorbani L, Ranjbar F, Noorazar G, Mosharkesh M: **Prevalence and risk factors of postoperative delirium in patients undergoing open heart surgery in northwest of iran**. *J Cardiovasc Thorac Res* 2013, **5**(3):97-99.

5. Brown CH: **Delirium in the cardiac surgical ICU**. *Curr Opin Anaesthesiol* 2014, **27**(2):117-122.

6. Leslie DL, Inouye SK: **The importance of delirium: economic and societal costs**. *J AM GERIATR SOC* 2011, **59 Suppl 2**:S241-S243.

7. Milbrandt EB, Deppen S, Harrison PL, Shintani AK, Speroff T, Stiles RA, Truman B, Bernard GR, Dittus RS, Ely EW: **Costs associated with delirium in mechanically ventilated patients**. *CRIT CARE MED* 2004, **32**(4):955-962.

8. Koster S, Hensens AG, van der Palen J: **The long-term cognitive and functional outcomes of postoperative delirium after cardiac surgery**. *ANN THORAC SURG* 2009, **87**(5):1469-1474.

9. Loponen P, Luther M, Wistbacka JO, Nissinen J, Sintonen H, Huhtala H, Tarkka MR: **Postoperative delirium and health related quality of life after coronary artery bypass grafting**. *SCAND CARDIOVASC J* 2008, **42**(5):337-344.

10. Martin BJ, Buth KJ, Arora RC, Baskett RJ: **Delirium: a cause for concern beyond the immediate postoperative period**. *ANN THORAC SURG* 2012, **93**(4):1114-1120.

11. Gottesman RF, Grega MA, Bailey MM, Pham LD, Zeger SL, Baumgartner WA, Selnes OA, McKhann GM: **Delirium after coronary artery bypass graft surgery and late mortality**. *ANN NEUROL* 2010, **67**(3):338-344.

12. Chan MT, Cheng BC, Lee TM, Gin T: **BIS-guided anesthesia decreases postoperative delirium and cognitive decline**. *J Neurosurg Anesthesiol* 2013, **25**(1):33-42.

13. Radtke FM, Franck M, Lendner J, Kruger S, Wernecke KD, Spies CD: **Monitoring depth of anaesthesia in a randomized trial decreases the rate of postoperative delirium but not postoperative cognitive dysfunction**. *Br J Anaesth* 2013, **110 Suppl 1**:i98-i105.

14. Plaschke K, Fichtenkamm P, Schramm C, Hauth S, Martin E, Verch M, Karck M, Kopitz J: **Early postoperative delirium after open-heart cardiac surgery is associated with decreased bispectral EEG and increased cortisol and interleukin-6**. *Intensive Care Med* 2010, **36**(12):2081-2089.

15. Cerejeira J, Batista P, Nogueira V, Vaz-Serra A, Mukaetova-Ladinska EB: **The stress response to surgery and postoperative delirium: evidence of hypothalamic-pituitary-adrenal axis hyperresponsiveness and decreased suppression of the GH/IGF-1 Axis**. *J Geriatr Psychiatry Neurol* 2013, **26**(3):185-194.

16. Tan MC, Felde A, Kuskowski M, Ward H, Kelly RF, Adabag AS, Dysken M: **Incidence and predictors of post-cardiotomy delirium**. *Am J Geriatr Psychiatry* 2008, **16**(7):575-583.

17. Kosar CM, Tabloski PA, Travison TG, Jones RN, Schmitt EM, Puelle MR, Inloes JB, Saczynski JS, Marcantonio ER, Meagher D *et al*: **EFFECT OF PREOPERATIVE PAIN AND DEPRESSIVE SYMPTOMS ON THE DEVELOPMENT OF POSTOPERATIVE DELIRIUM**. *Lancet Psychiatry* 2014, **1**(6):431-436.

18. Leung JM, Sands LP, Lim E, Tsai TL, Kinjo S: **Does preoperative risk for delirium moderate the effects of postoperative pain and opiate use on postoperative delirium?** *Am J Geriatr Psychiatry* 2013, **21**(10):946-956.

19. Burkhart CS, Dell-Kuster S, Gamberini M, Moeckli A, Grapow M, Filipovic M, Seeberger MD, Monsch AU, Strebel SP, Steiner LA: **Modifiable and nonmodifiable risk factors for postoperative delirium after cardiac surgery with cardiopulmonary bypass**. *J Cardiothorac Vasc Anesth* 2010, **24**(4):555-559.

20. Norkienė I, Ringaitienė D, Kuzminskaitė V, Šipylaitė J: **Incidence and Risk Factors of Early Delirium after Cardiac Surgery**. *BIOMED RES INT* 2013, **2013**:1-5.

21. Smulter N, Lingehall HC, Gustafson Y, Olofsson B, Engstrom KG: **Delirium after cardiac surgery: incidence and risk factors**. *INTERACT CARDIOV TH* 2013, **17**(5):790-796.

22. Fitzgerald JM, Adamis D, Trzepacz PT, O'Regan N, Timmons S, Dunne C, Meagher DJ: **Delirium: a disturbance of circadian integrity?** *MED HYPOTHESES* 2013, **81**(4):568-576.

23. Trompeo AC, Vidi Y, Locane MD, Braghiroli A, Mascia L, Bosma K, Ranieri VM: **Sleep disturbances in the critically ill patients: role of delirium and sedative agents**. *MINERVA ANESTESIOL* 2011, **77**(6):604-612.

24. Gerresheim G, Schwemmer U: **[Dexmedetomidine]**. *ANAESTHESIST* 2013, **62**(8):661-674.

25. Harsoor SS, Rani DD, Lathashree S, Nethra SS, Sudheesh K: **Effect of intraoperative Dexmedetomidine infusion on Sevoflurane requirement and blood glucose levels during entropy-guided general anesthesia**. *J Anaesthesiol Clin Pharmacol* 2014, **30**(1):25-30.

26. Naguib AN, Tobias JD, Hall MW, Cismowski MJ, Miao Y, Barry N, Preston T, Galantowicz M, Hoffman TM: **The role of different anesthetic techniques in altering the stress response during cardiac surgery in children: a prospective, double-blinded, and randomized study**. *Pediatr Crit Care Med* 2013, **14**(5):481-490.

27. Wang Y, Xu X, Liu H, Ji F: **Effects of dexmedetomidine on patients undergoing radical gastrectomy**. *J SURG RES* 2015, **194**(1):147-153.

28. Abd AN, Chue MC, Yong CY, Hassan Y, Awaisu A, Hassan J, Kamarulzaman MH: **Efficacy and safety of dexmedetomidine versus morphine in post-operative cardiac surgery patients**. *Int J Clin Pharm* 2011, **33**(2):150-154.

29. Barletta JF, Miedema SL, Wiseman D, Heiser JC, McAllen KJ: **Impact of dexmedetomidine on analgesic requirements in patients after cardiac surgery in a fast-track recovery room setting**. *PHARMACOTHERAPY* 2009, **29**(12):1427-1432.

30. Maldonado JR, Wysong A, van der Starre PJ, Block T, Miller C, Reitz BA: **Dexmedetomidine and the reduction of postoperative delirium after cardiac surgery**. *PSYCHOSOMATICS* 2009, **50**(3):206-217.

31. Ji F, Li Z, Young N, Moore P, Liu H: **Perioperative dexmedetomidine improves mortality in patients undergoing coronary artery bypass surgery**. *J Cardiothorac Vasc Anesth* 2014, **28**(2):267-273.

32. Curtis JA, Hollinger MK, Jain HB: **Propofol-based versus dexmedetomidine-based sedation in cardiac surgery patients**. *J Cardiothorac Vasc Anesth* 2013, **27**(6):1289-1294.

33. Dasta JF, Jacobi J, Sesti AM, McLaughlin TP: **Addition of dexmedetomidine to standard sedation regimens after cardiac surgery: an outcomes analysis**. *PHARMACOTHERAPY* 2006, **26**(6):798-805.

34. Lin YY, He B, Chen J, Wang ZN: **Can dexmedetomidine be a safe and efficacious sedative agent in post-cardiac surgery patients? a meta-analysis**. *CRIT CARE* 2012, **16**(5):R169.
